# Development of a pooled probe method for locating small gene families in a physical map of soybean using stress related paralogues and a BAC minimum tile path

**DOI:** 10.1186/1746-4811-2-20

**Published:** 2006-12-08

**Authors:** Kay L Shopinski, Muhammad J Iqbal, Jeffry L Shultz, Dheepakkumaran Jayaraman, David A Lightfoot

**Affiliations:** 1 Institute for Sustainable and Renewable Resources (ISRR), Institute for Advanced Learning and Research (IALR), Danville, VA 24540, USA; 2 Department of Plant, Soil and Agriculture Systems, Room 176, Agriculture Building, MC 4415, Southern Illinois University, Carbondale, IL 62901, USA; 3 Dept of Plant Molecular Biology, United States Department of Agriculture, Peoria, IL, USA; 4 Dept of Soybean Genetics, United States Department of Agriculture, Stoneville, MS 38776, USA

## Abstract

**Background:**

Genome analysis of soybean (*Glycine max *L.) has been complicated by its paleo-autopolyploid nature and conserved homeologous regions. Landmarks of expressed sequence tags (ESTs) located within a minimum tile path (MTP) of contiguous (contig) bacterial artificial chromosome (BAC) clones or radiation hybrid set can identify stress and defense related gene rich regions in the genome. A physical map of about 2,800 contigs and MTPs of 8,064 BAC clones encompass the soybean genome. That genome is being sequenced by whole genome shotgun methods so that reliable estimates of gene family size and gene locations will provide a useful tool for finishing. The aims here were to develop methods to anchor plant defense- and stress-related gene paralogues on the MTP derived from the soybean physical map, to identify gene rich regions and to correlate those with QTL for disease resistance.

**Results:**

The probes included 143 ESTs from a root library selected by subtractive hybridization from a multiply disease resistant soybean cultivar 'Forrest' 14 days after inoculation with *Fusarium solani *f. sp. *glycines *(*F. virguliforme*). Another 166 probes were chosen from a root EST library (Gm-r1021) prepared from a non-inoculated soybean cultivar 'Williams 82' based on their homology to the known defense and stress related genes. Twelve and thirteen pooled EST probes were hybridized to high-density colony arrays of MTP BAC clones from the cv. 'Forrest' genome. The EST pools located 613 paralogues for 201 of the 309 probes used (range 1–13 per functional probe). One hundred BAC clones contained more than one kind of paralogue. Many more BACs (246) contained a single paralogue of one of the 201 probes detectable gene families. ESTs were anchored on soybean linkage groups A1, B1, C2, E, D1a+Q, G, I, M, H, and O.

**Conclusion:**

Estimates of gene family sizes were more similar to those made by Southern hybridization than by bioinformatics inferences from EST collections. When compared to *Arabidopsis thaliana *there were more 2 and 4 member paralogue families reflecting the diploidized-tetraploid nature of the soybean genome. However there were fewer families with 5 or more genes and the same number of single genes. Therefore the method can identify evolutionary patterns such as massively extensive selective gene loss or rapid divergence to regenerate the unique genes in some families.

## Background

Soybean (*Glycine max *(L.) Merr.) genome has a tetraploid origin with 20 consensus linkage groups representing 20 pairs of chromosomes with a genome size of 1.115 Gbp [1,2]. Within the soybean chromosomes there were large regions of euchromatic and heterochromatic DNA. Two separate duplications or hybridizations in soybean progenitor genomes were hypothesized to have occurred [3,4]. Homeologous regions abound conserved synteny among regions ranges from not detectable (diploidized) to highly conserved (tetraploid to octaploid; [4-7]. Gene rich and gene poor regions exist [5,8] but have not been correlated with euchromatin or homeologous regions to date.

Physical maps provide estimates of relationships between loci, genes and regions of chromosomes at the base pair (bp) scale [4,5,9,10]. Cloned sections of genomic DNA can be aligned in an ordered, contiguous, overlapping arrays or contigs. The minimum tiling paths (MTP) or best coverage paths (BCP) have been developed by choosing clones from within contigs [6,11]. An interactive soybean physical map [5,6,12] is represented through the Soybean Genome Database (SGD) [5]. The soybean physical map was constructed from 72,942 clones anchored with 404 microsatellite and RFLP markers that detected multiple homologues, 13,747 BAC end sequences (BES) and 1,053 anchoring site-specific BES derived microsatellite markers. In build 2 and build 3 of the soybean physical map, there were 69,684 clones encompassing 8.7 haploid genomes in 5,597 contigs (build 2) that were merged to 2,905 contigs (build 3). One minimum tile developed for build 2 and build 3 was called MTP2BH and used 8,064 clones that encompassed 1.09 Gbp [5,6,10]. In build 4 there were 42,000 clones in 2,854 contigs (6 fold coverage of the genome). The MTP of build 4 encompassed 4,224 clones covering 0.79 Gbp [5-7] because conserved homeologous regions were tiled once.

Southern hybridizations with ESTs can locate genes on physical maps to generate gene paralogue maps [13]. EST based gene maps have been made for many plant species; *Zea mays *[14], *Medicago truncatulata *[15], and *Glycine max *[16,17]. EST probes have the advantage of hybridization to all the conserved members of their gene families, functionally those sharing more than about 75% sequence identity [13]. Short oligomeric overgo probes [18,19] have provided high-throughput for EST mapping. Overgo probes were designed to be specific to a single paralogue but many were prone to false hybridizations [15,20-22] especially in soybean [22,23]. Other methods that have been used for physical mapping include in-situ hybridization (FISH) and chromosome landmarks in plants and animals [15,24,25]. In order to anchor unknown ESTs in sorghum physical map, [26] immobilized BAC DNA in tubes and identified and sequenced unknown cDNAs that hybridize to the immobilized DNA. However, in this study we used ESTs that had homology to known genes and identified their locations in the soybean physical map.

Genes involved in plant defense, stress response, secondary metabolism and signal transduction were differentially regulated in response to *Fusarium solani *f. sp. *glycines *(Fsg) infection [27,28]. Fsg (also called *F. virguliforme*) is the causative agent of sudden death syndrome (SDS) of soybean [29,30]. Earlier studies [31,32] identified six QTL that underlie resistance to SDS in a segregating population. Multi-locus resistance to SDS suggests a complex response to the disease by the plant and the involvement of a large number of genes in response to the fungal pathogen. The identification of the location of the ESTs representing defense related genes may show the genomic distribution of SDS response related gene rich regions. Candidate gene association with the QTL for resistance to SDS may be tested in the soybean genome.

ESTs have been used to identify single nucleotide polymorphism (SNP) or restriction fragment length polymorphism (RFLP) and were located in the soybean genetic map [24,33,34]. The polymorphism identified by using different restriction enzymes ranged from 18–50% of the cDNA clones [33] and less than one third of EST clusters [24]. However, placement of ESTs by physical map location is not dependent on polymorphism providing efficiency to the endeavor. There were 962 QTL for disease resistance and agronomic traits listed at Soybase [1]. Defense and stress-related ESTs physical map locations may provide candidate genes underlying many QTL not just SDS.

## Materials and methods

The two BAC libraries used were created from soybean cv 'Forrest' using the restriction enzymes *Hind *III and *Bam *HI [12,35]. The clones were annotated with initials as H for a *Hind *III clone and B for a *Bam *HI clone.

### Preparation of high density membranes containing minimum tiling path (MTP) BAC clones

The minimum tiling path (MTP) of build 2 was developed at Southern Illinois University, Carbondale, IL [5,6] and can be viewed through the soybean genome database (SoyGD). The soybean physical map was constructed from 69,684 clones encompassing 8.7 haploid genomes that were merged to 2,953 contigs. There were 8,064 clones in the MTP2BH that encompassed ~1-fold coverage of the soybean genome, or about 1.09 Gbp [5-7]. The selected BAC clones were spotted on Amersham Hybond N^+ ^nylon membrane using a robot and a 384 pin head (Flexys^® ^robot, Genomic Solutions, Ann Arbor, MI) in duplicate. The membrane was placed on the Luria-Bertani (LB) agar containing 15 mg/mL tetracycline (Sigma Aldrich Co., St. Louis, MO) and incubated at 37°C for 12 h after spotting. The membranes were processed according to [36,37].

### Selection of EST probes

The two sets of ESTs used in the study were selected from two different cDNA libraries. The first set was selected from a soybean variety 'Forrest' root library (FiS library) enriched for genes that were expressed in response to Fsg inoculation [27,28]. The second set of ESTs was selected based on their homology to the known plant defense and stress related genes from a soybean variety 'Williams' root library (Gm-r1021 library) obtained from Research Genetics Inc. [38].

### Preparation of EST probes

Plasmid DNA carrying the EST insert were isolated [27], treated with RNase and restriction digested with *Bst *ZI (FiS library) or with *Xho *I and *Eco*RI (Gm-r1021 library). In cases where good restriction was not accomplished, the insert were amplified by PCR using T7 and T3 universal primers. The restriction digested or PCR amplified inserts were electrophoresed on 1% (w/v) agarose gel and insert DNA bands were purified by Zymoclean Gel DNA Recovery Kit (Zymo Research Corp, Orange, CA). DNA concentrations were measured by BioPhotometer 6131 for the FiS library or approximated from band intensities on gels for the Gm-r1021 library.

The samples were arranged in a 12 × 12 grid for the FiS library and 13 × 13 grid for the Gm-r1021 library in order to develop horizontal row and vertical column pools. The FiS library contained one blank sample (143 ESTs) and the Gm-r1021 library contained 3 blank samples (166 ESTs). These blank samples were replaced with water in the pools. Equal amounts of DNA were combined to make pools. The volume of pooled DNA was adjusted to 45 μl with dH_2_O. The mixture was then denatured at 95°C for 4–5 min and cooled immediately on ice for 2 min. The denatured DNA was added to a Ready-To-Go DNA Labeling beads (Amersham Biosciences UK Limited, Little Chalfont, Buckinghamshire, England) and 5 μl of 6000 Ci/mmol α^32^P dCTP was added and incubated at room temperature for 30 min. The labeled probe was diluted with 20 μl of dH_2_O and passed through a Sephadex G-50 column at 6000 g for 5 min to remove unincorporated radio nucleotides.

### Colony pre-hybridization, hybridization and post hybridization washes and exposing film to hybridized membranes

The MTP membrane was saturated with 2X SSC and pre-hybridized in 5 X Denhardt buffer, 1% (w/v) SDS, 6X SSC, denatured pCDL04541 vector DNA (GenBank No. 184978) at 65°C for 2 h. The probe pools were denatured and added to the hybridization tube. The membrane was hybridized for approximately 21 h at 65°C (Tm-30 C assuming 50 % GC content and probes > 200 bp). The membrane was washed twice with pre-warmed (65°C) wash solution (2X SSC, 0.1% (w/v) SDS) at 65°C for 10 min with continuous agitation. The membrane was washed with pre-warmed (65°C) higher stringency (Tm-25C) solution (1X SSC, 0.1% (w/v) SDS) at 65°C for 10 min with continuous agitation. The membranes were checked for activity using a Geiger counter and the last wash step was repeated if needed.

The membranes were placed in cellophane wrap and sides were sealed with a food sealer. The membranes were used to expose Kodak BioMax MR film (Fisher Scientific Co., Fair Lawn, NJ) for 24 h or used to expose a bleached PhosphorImage cassette. The film were developed in 20% (v/v) Kodak GBX developer solution, and 20% (v/v) Kodak GBX replenishing solution (Fisher Scientific Co., Fair Lawn, NJ) for 3 min each. The exposed PhosphorImage cassettes were developed using a PhosphorImager 445SI scanner (Molecular Dynamics, Inc. Sunnyvale, CA) and scanned by using scanner control (version 3.51) at 176 micron resolution. The image analysis software Image QuaNT™ version 4.1 (Molecular Dynamics, Inc. Sunyvale, CA) was used to visualize the images.

### Southern hybridizations to restriction digest of BAC DNA

Southern hybridizations were performed on select EST/BAC hybridization combinations. The BAC DNA was restriction digested using the corresponding restriction enzymes (*Hind *III or *Bam *HI) that were used to make the libraries. BAC DNA was extracted by alkaline lysis method and 2 μg DNA was digested with the 1 μl of restriction enzyme for 20 h. The entire sample was electrophoresed in a 1% (w/v) agarose gel at 60 volts for approximately 16 h. The DNA from the gel was transferred to Hybond N^+ ^membrane by neutral transfer protocol for 20 h according to the instructions provided with the membrane (Amersham Pharmacia Biotech Limited, Buckinghamshire, England). The DNA was UV cross-linked to immobilize on the membrane. The probes were prepared as described earlier except that instead of pools, only single ESTs were labeled. Pre-hybridization, hybridization, and washes were carried out as above. The PhosphorImager was used to expose the cassette and acquire data.

### Data analysis

The images generated from the initial pool hybridizations were scored based on the ability of the EST pool to hybridize to the duplicate clones on the membrane. The address of the EST's within the horizontal by vertical grid provided the means to identify the single EST responsible for the clone positive. The data was entered into two spreadsheets. The first was G-browse version 3 (derived from version 2 by manual merges [12,38]; and the second was version 4 (a rebuild at high stringency) [5,6]; of the soybean physical map. In both builds contigs and singleton clones that were not yet anchored to linkage groups were placed in a single large pseudo-linkage group called Queue. Clones removed from contigs at the high stringencies used for build 4 can be reinserted to the most likely build 4 contig by inference from the overlapped clones in FPC. By this method locations for almost all clones may be inferred within the build 4 map. Many clones were located on a major linkage group (MLG) in build 3 were moved to Queue in build 4. Some Queue contigs can be located on the map by merges, by examination of the nascent build 5, by genetic linkages provided by BES [39] or by examination of the whole genome shotgun sequence to be released by DOE in 2007.

## Results

### Paralogue clusters were inferred with EST probes from the FiS library

Genes or sequences were paralogous if they were derived from a duplication event and were present within the same species. Here, soybean ESTs were hybridized to soybean BAC clones from an MTP with minimally overlapped clones. Therefore, multiple hybridizations were considered to be the consequence of detecting paralogues at different locations in the genome. From the total 143 EST probes, 101 hybridized to BAC clones on the MTP membrane while the remaining 42 probes provided only weak signals in one or both pools and were not scored (Table 1) [see Additional file 1]. The 101 EST probes hybridized to 334 putative paralogues. The putative paralogues were distributed among 216 colonies (BAC clones) because 58 BAC clones contained putative paralogues to more than one EST (mean 2.15 per BAC; Table 2). The number of EST probes that hybridized per BAC clone ranged from 1 to 12. The BAC clones that located a single EST (158) were in the majority (73%). There were 54 BAC clones that hybridized with 2–4 ESTs. There were 4 BAC clones that were inferred to contain 5–12 different EST paralogues (Table 2).

**Table 1:** Identification of the number of hybridizing colonies (BAC clones) and inferred significantly conserved paralogues (G) identified by probes (P) to 201 small gene families in soybean. MTP BAC DNA was hybridized to 166 EST probes from cv. Williams (Gm library) and 143 EST probes from cv. Forrest (Fi library). Hybridization stringency was about 25 C below Tm so paralogue detected were expected to share more than 75% nucleotide identity.

	Probe	Number of paralogues per gene family											
		
Library	total	1	2	3	4	5	6	7	8	9	10	13	15
**Fi-P**	**101**	**34**	**20**	**14**	**9**	**5**	**5**	**5**	**2**	**2**	**3**	**1**	**1**
Fi-G	334	34	40	42	36	25	30	35	16	18	30	13	15
**Gm-P**	**100**	**36**	**29**	**7**	**12**	**4**	**5**	**2**	**2**	**0**	**1**	**2**	**0**
Gm-G	279	36	58	21	48	20	30	14	16	0	10	26	0

**Total-P**	**201**	**70**	**49**	**21**	**21**	**9**	**10**	**7**	**4**	**2**	**4**	**3**	**1**
Total-G	613	70	98	63	84	45	60	49	32	18	40	39	15

**Table 2:** Identification of the number of EST clusters within individual BACs(G) identified by probes to 201 small gene families in soybean MTP BAC DNA using soybean 166 cv. Williams ESTs (Gm library) and 143 cv. Forrest ESTs (Fi library).

Probe		Number of different gene families per BAC													
		
Library	total	1	2	3	4	5	6	7	8	9	12	14	15	21	39
Fi	216	158	36	6	12	0	1	1	0	1	1	0	0	0	0
Gm	130	100	15	2	5	1	1	0	1	0	0	1	2	1	1
**Pooled**	334*	246*	63*	**8**	**17**	**1**	**2**	**1**	**1**	**1**	**1**	**1**	**2**	**1**	**1**

* 12 BACs hybridized to 2 different probes from 2 different libraries

### Paralogous gene family sizes were inferred with EST probes from the FiS library

Each BAC clone that hybridized to an EST and formed part of a separate contig was inferred to contain a paralogue of that gene family. The number of paralogues inferred per EST ranged from 1 to 15 (Table 1) [see Additional file 2]. There were 34 ESTs (~34%) that hybridized to one BAC clone that may be single copy or highly diverged gene families. More ESTs hybridized to 2 and 4 BACs in soybean than to 3 BACs. Comparison with the diploidized *A. thaliana *genome (Table 3) [40] suggested the trend was significant and might be expected for a paleo-polyploid genome with conserved tetraploid and octoploid regions. The multi-copy (6–15 copies) paralogues included elongation factor 1B alpha-subunit, two un-annotated ESTs, the 5.8S, 18S and 25S ribosomal RNA cluster, a putative water channel protein, an ascorbate peroxidase 1, and a lipoxygenase (Table 4) all known multi-locus gene families in soybean [38].

**Table 3:** Proportion of genes in different organisms present as either singletons or in paralogous families.

Species	No of genes*	Unique gene families containing					
		
		1	2	3	4	5	> 5 member
*H. influenzae*	1,587	88.8%	6.8%	2.3%	0.7%	0.0%	1.4%
*S. cerevisiae*	5,105	71.4%	13.8%	3.5%	2.2%	0.7%	8.4%
*D. melanogaster*	10,736	72.5%	8.5%	3.4%	1.9%	1.6%	12.1%
*C. elegans*	14,177	55.2%	12.0%	4.5%	2.7%	1.6%	24.0%
*Arabidopsis*	11,601	**35.0**%	12.5%	7.0%	4.4%	3.6%	**37.4**%
*G.max *(soybean)	201	**35.0**%	**25.0 %**	10.4%	**10.4%**	4.5%	15%

* The number of genes in the genomes of *Haemophilus influenzae, S. cerevisiae, Drosophila, C. elegans, Arabidopsis *and *Glycine max *that were present either as singletons or in gene families with two or more members were listed. To be grouped in a gene family, two genes had to show similarity exceeding a BLASTP value E,10 -20 and a FASTA alignment over at least 80%of the protein length or hybridize at 65 C and 0.1 M ionic strength (Tm -25). In column 1, the number of genes that were unique plus the number of gene families were listed. Columns 2 to 6 give the percentage of genes present as singletons or in gene families of n members (from TAIR 2000).

**Table 4:** High copy number gene families detected in the MTP with EST probes. When an EST hybridize to a BAC clone on the MTP membrane, it is considered as positive for that EST.

GenBank accession	Homology to the known genes	No. of BACs + ve for EST	EST source
AI437902	Threonine synthase	7	Gm
AI442296	Calmodulin like protein	7	Gm
AI441021	Calcium dependent protein kinase	8	Gm
AI460618	Calmodulin-stimulated calcium ATPase	8	Gm
BI273655	Ascorbate peroxidase type 1	8	Fi
BI273688	Ascorbate peroxidase type 2	8	Fi
BI347330	Putative elongation factor 1B alpha-subunit	9	Fi
BI273660	EST	9	Fi
CF675620	5.8S, 18S and 25S ribosomal RNA	10	Fi
BI119568	EST	10	Fi
BI119558	Putative water channel protein	10	Fi
AI440721	MAP kinase kinase alpha protein kinase	10	Gm
AI460671	Kinesin like protein A	13	Gm
AI441809	β-galactosidase.	13	Gm
BI119573	Ascorbate peroxidase type 3	13	Fi
BI273669	Lipoxygenase	15	Fi

### Paralogue clusters were inferred with EST probes from the Gm-r1021 library

From the total 166 EST probes, 100 hybridized to BAC clones on the MTP membrane, the remaining 66 provided only weak signals and were not scored (Table 1) [see Additional files 1 and 3]. The 100 useful EST probes hybridized to 279 putative paralogues distributed among 130 BAC clones that were inferred to contain paralogues (Table 2). One hundred of the BAC clones contained a single paralogue. Twenty-two BAC clones contained sequences that may have been paralogous to 2–4 probes. Eight BAC clones contained clusters of more than 5 different paralogues and may have gene rich regions. The number of ESTs hybridized per BAC clone ranged from 1 to 39.

### Paralogous gene family sizes were inferred with EST probes from the Gm-r1021 library

The number of paralogues per EST (BAC clones that hybridize to an EST probe) ranged from 1 to 13 (Table 1) [see Additional files 1, 3 and 4]. There were 36 ESTs (36%) that hybridized to one BAC clone. Significantly more ESTs hybridized to 2 and 4 BACs than 3 BACs. Comparison to gene family size in *A. thaliana *[40] again suggested the 2 and 4 member gene families were a feature of conserved tetraploid and octoploid regions in the soybean genome (Table 3). The multi-copy (6–13 copies) paralogues includedthreonine synthase, calmodulin like protein, two distinct calcium dependent protein kinases, calmodulin-stimulated calcium ATPase, MAP kinase kinase alpha protein kinase, kinesin like protein A and β-galactosidase. All were known multi-gene families [38].

### Summary of paralogue clusters and gene family sizes from both libraries

Common trends within the data for each library suggested FiS and the Gm-r1021 data be combined for further analysis. Twelve BAC clones (0.15% of the clones in the MTP2BH) contained EST probes from both libraries (Table 2). The 12 clones was more than expected since only about 1.5 % of BAC clones hybridized to at least one of the probes used per library. All the EST probes were non-redundant. From the total 309 EST probes, 201 hybridized to colonies containing BAC clones (Table 1) from two pools. There were 613 colony hybridizations with the 201 probes indicating the presence of homologous sequences on the clones. However, 100BAC clones contained more than one EST. Therefore, the ESTs were located to 346 BAC clones (Table 2). The BAC clones that located a single EST probe were in the majority (246 or 73.6%). The BAC clones with 2–4 ESTs accounted for 26 % (88) of the total that hybridized. The BAC clones with 5–12 EST clusters accounted for 3.6 % (12) of the total BAC clones with 4 as mode. The gene rich clones were potential candidate for sequencing.

### Confirmation of MTP hybridizations by Southern hybridization

**Figure 1:**
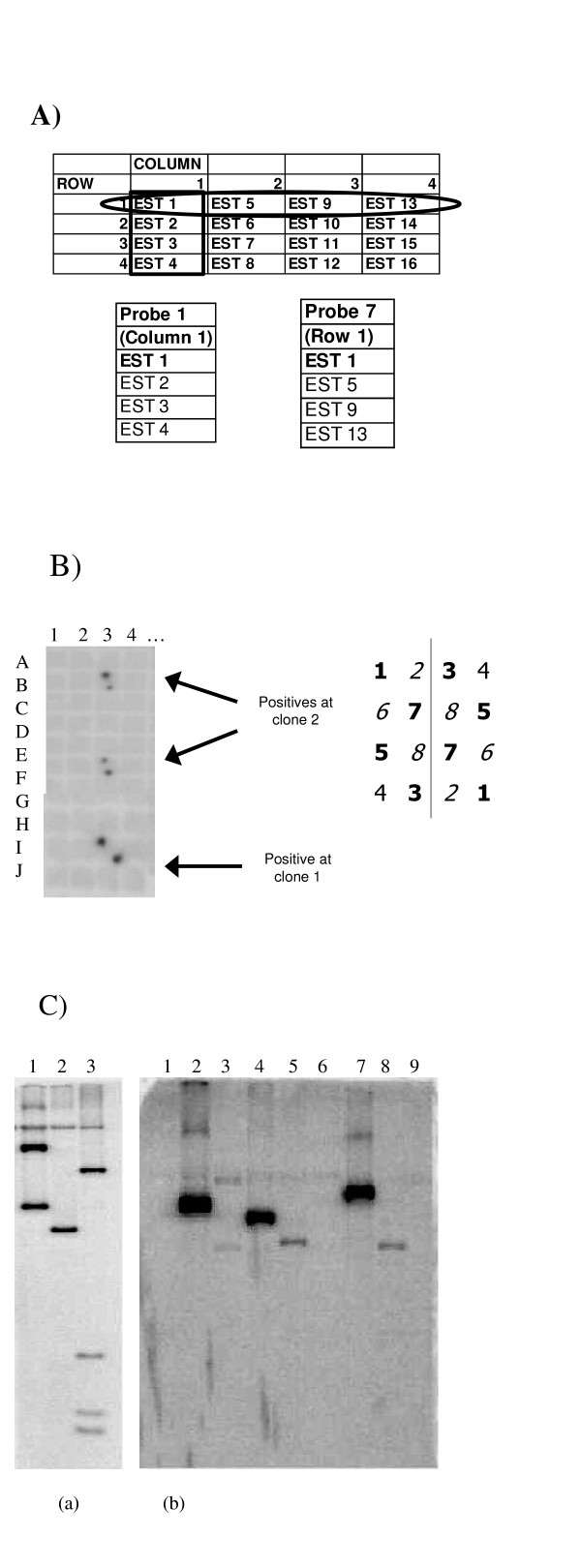
Methods for identifying gene families in the minimum tile path of BAC clones. Panel A): The strategy for probe pool designs. Panel B): An example of three positive clones from colony hybridizations to double spotted colonies from four 384 plates per location. Panel C): DNA from BAC clones identified from the MTP hybridizations were restriction digested with the restriction enzyme used for insertion, transferred to a membrane and hybridized to the EST probe. (a): The BAC clones B48B23, H59N09, and H76L07 (Lane 1, 2, and 3) were positive for the identified EST FiS1H9 (BI273631 a histone H2A orthologue) from the initial MTP hybridization. (b): DNA of BAC clones B23A05, B23C13, B38M08, B48M07, H15A05, H15A06, H20G14, H36D08, and H42K03 (Lane 1, 2, 3, 4, 5, 6, 7, 8, and 9 respectively) were hybridized to the EST Fi36H18 (BI347330 a translational elongation factor 1B-alpha 1 orthologue) identified from the initial MTP hybridization for second confirmation. The hybridization proved that 6 out of the 9 (Lane 2, 3, 4, 5, 7, and 8) initial positives were positive with a second Southern hybridization.

Moderately stringent conditions (Tm -25 C) were used in hybridization of EST probe pools to the membrane containing a set of BAC clones representing MTP set. Each BAC clone was duplicated on the membrane and each probe (an EST) was hybridized twice; i.e. once in vertical and once in horizontal pool. The BAC clones were considered to contain the hybridizing EST only if both the duplicate clones on the membrane hybridize consistently in horizontal and vertical probe pools. In order to further validate our data, a number of BAC clones that were positive in MTP membrane hybridizations were reconfirmed by independent Southern hybridizations to BAC DNA (Fig. 1 Panel (C)). Clear differences between band sizes of the paralogues present on separate BAC clones were observed. The majority (11/16) of clones that hybridized as colonies also hybridized to bands in Southerns made from digested BACs and gel derived membranes (Table 5). There was correlation between numbers of colony hybridization positives with that of the second confirmation. An EST with similarity to a translational elongation factor 1B-alpha 1 with nine MTP membrane hybridization positives had 6 paralogues confirmed by the second Southern hybridization. However, another EST (BI273631) that encoded a protein with similarity (96%) to histone H2A with 3 colony hybridization positives had only one paralogue confirmed by the second Southern hybridization. Since colony positives derived from 4 spots from 2 filters the negatives may have derived from the 2–10% of clones that were contaminated [7] miss-identified or clones that spontaneously deleted part of the insert. The DNA was made from a single colony per BAC, in retrospect sampling multiple clones per BAC location would have been wiser.

**Table 5:** Correspondence between paralogue copy number A: Estimated from published Southern hybridizations and the colony hybridizations to the MTP. B: Comparison of paralogue number in MTP colony hybridization and by Southern hybridization.

Probe/gene family	Southern estimate of copy number	Reference	MTP estimate of copy number
A:			
G-box factor	5–7	Hong, et al. (1995)	1
Epoxide hydrolase	5	Arahira et al. (2000)	1
Chalcone synthase	3–7	Estabrook et al., (1991)	1
Phenylalanine ammonia lyase	2–3	Estabrook et al., (1991)	1
ATP synthase	2–3	Smith et al. (1994)	3
Aspartate aminotransferase 1	1–2	Gebhardt et al. (1998)	3
Leghemoglobin.	2	Ji et al. (1994)	3
4-coumarate CoA ligase 1.	1–3	Lindermayr et al. (2002)	5
Calmodulin	4	Lee et al. (1995)	5
Nodulin 22	4–5	Sandal et al. (1987)	5
B:			
EST BM499228	1	This work	1
EST BI347333	1	This work	3
EST BI273631	3	This work	3
EST BI347330	6	This work	9

### Distribution of BAC clones and ESTs in the soybean physical map

The BAC clones that hybridized to the EST probes were searched on G-Browse at SoyGD [5]. Build 3 was made by merging contigs from build 2. Therefore, build 3 and the newer version, build 4 were both used to locate the BAC clones in the soybean physical map.

### Build 3 locations

The 8,064 BAC clones of MTP2BH were located in 2,905 contigs with about 3 clones from each contig (range 1–9). The number of BAC clones from within a single contig that had hybridized to different EST probes ranged from 1 to 4 [see Additional file 4]. The 346 BAC clones that contained ESTs were assembled in 218 contigs. There were 62 marker anchored contigs and 156 contigs not yet assigned to linkage groups, placed in Queue. The ESTs bearing contigs that were located on a linkage group in the physical map encompassed 25 Mbp and in Queue a further 75 Mbp. The regions of the contigs were overlapped by 32 QTL for disease or stress related traits. The number of ESTs per contig showed that 164 contigs had only one EST paralogue. However, clusters of different paralogues were located on 54 contigs (42 had 2, 2 had 3, 9 had 4, 1 had 8). One hundred and thirty-four contig sized clusters of ESTs were present on 17 different linkage groups according to build 3 data. However, this may be overestimated because build 3 contigs contain merges that were not supported in build 4.

**Figure 2:**
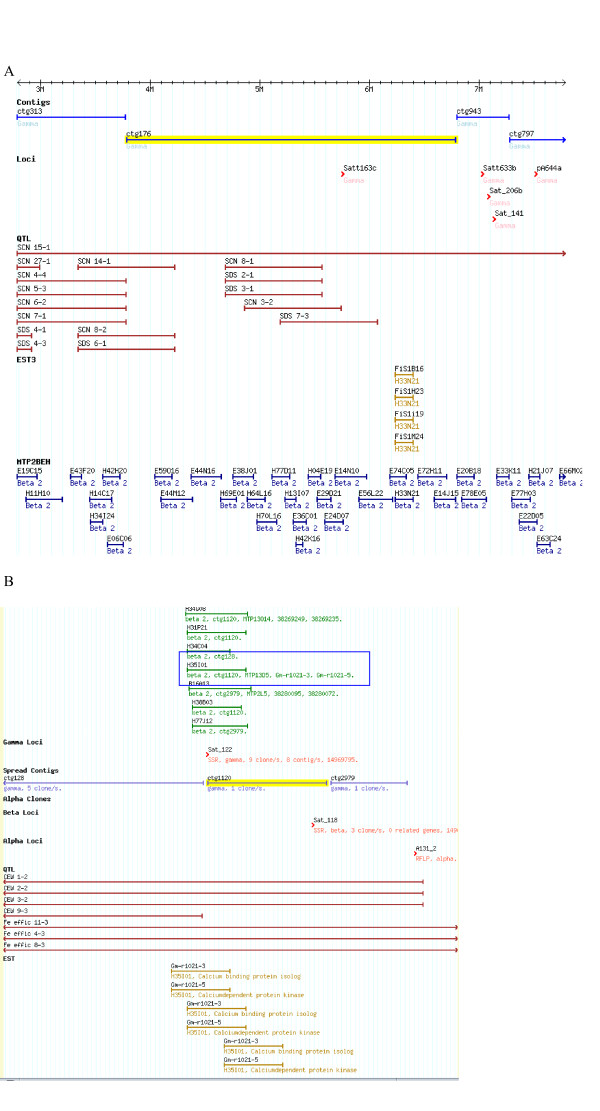
SoyGD images showing the locations of ESTs in build 3. Panel A): A 'Forrest' physical map of Build 3 contig176 showing BAC clones, QTL and markers. The contig is located on linkage group G and contains SSR marker Sat_163. The BAC clone was positive for ESTs encoding a QM-family orthologue, two types of ascorbate oxidase (laccase, or diphenol oxidase paralogues) and an un-annotated EST. Panel B): A 'Forrest' physical map contig 1120 representing BAC clone H35I01. This contig was located on MLG H and contained SSR marker Sat_122. The BAC clone was positive for ESTs BI119568 and AI441021 homologous to calcium binding protein isolog and calcium dependent protein kinase.

Some contigs had clusters of gene paralogues that were of related function or in related pathways. Among the more interesting candidate genes was the QM-family orthologue found on linkage group [41] that was clustered with two types of ascorbate oxidase (laccase, or diphenol oxidase paralogues) and an un-named EST on a build 3 contig (ctg176; Figure 2A). The contig encompassed 18 clones of MTP2 and measured around 3 Mbp. However, the contig 'ctg176' was placed in Queue by build 4 as cgt3198. Because the map positions of build 3 were not reliable [5], analysis of candidate genes was concentrated on build 4 hereafter.

### Build 4 locations

Many clones were removed from contigs during the editing process of Version 4 so that fewer EST hybridizing clones were included in that build. The 8,064 BAC clones of MTP2BH were located in 2,854 contigs with about 2 clones from each contig (range 1–6). The number of BAC clones from a single contig that hybridized to an EST ranged from 1 to 3 (Supplemental Table 1-3). In build 4, 131 contigs were identified with ESTs and 95 of the contigs were in queue. ESTs were located on 36 contigs on the physical map. Queue contigs encompassed 48 Mbp and anchored contigs encompassed 16 Mbp. The regions of the contigs were overlapped by 42 QTL for disease or stress related traits. Among the map anchored contigs, 30 contained a single BAC clone with a paralogue or paralogue cluster, five contigs contained 2 BAC clones with a paralogue or paralogue cluster and one contig contained 3 BAC clones with a paralogue or paralogue cluster. Similarly, the number of ESTs per contig showed 92 contigs had only one EST paralogue. However paralogue clusters were located to 38 contigs (21 had 2, 8 had 3, 5 had 4, 1 had 5, 1 had six, 1 had 7 and 1 had 15).

Contigs had clustered gene paralogues of related function or in related pathways. The contig 1751 (in queue) had a cluster of 15 ESTs paralogues that included 4-coumarate CoA ligase isoform-2, 7-O-methyltransferase, β-galactosidase, calmodulin, calmodulin-like protein, calmodulin-stimulated calcium ATPase and MAP kinase kinase alpha protein kinase. Contig 1120 that had EST homologous to calcium binding protein isolog and calcium-dependent protein kinase, that were located on major linkage group H by the SSR marker Sat_122 (Fig. 2). Data also showed 32 ESTs with paralogues located on two or more contigs within the genome. The EST BI347339, homologous with *G. max *myo-inositol-1-phosphate synthase, was identified on two locations within the genome. Another EST, BI119573, homologous to *G. max *ascorbate peroxidase, was identified at 5 different locations within the genome. One location was on MLG D1AQ, within contig 9088, that contained SSR marker Satt482.

Twenty-three of clusters ESTs were located to 11 different MLG of build 4 to date. Six EST clusters were mapped to MLG A1, two were mapped to B1, one to B2, 5 mapped to C2, 10 mapped to D1AQ, 4 mapped to E, 12 mapped to G, 2 mapped to I, 3 mapped to M, 3 mapped to H, and another 3 mapped to MLG O. Considering clones present in build 3 that were represented only by an overlapping clone in build 4 did not increase the number of contigs placed.

## Discussion

The estimated physical locations of gene paralogues within the physical map can provide a tool for understanding the genetic architecture of plants [13,15,42]. Contig associations located the approximate position of a number of plant defense and stress related ESTs (genes) on the soybean physical map build 3 and 4, in SoyGD [5]. The placement of BACs that hybridized to a common probe into separate contigs allowed the inference that separate paralogues had been detected.

Sixty-five percent of the ESTs used in this experiment hybridized to at least one BAC clone in two pools. Of the probes that so hybridized, 35% appeared to detect a single paralogue in the genome i.e. they hybridize to one BAC clone on the MTP set, unexpected for a paleo-tetraploid [4-7]. The low hybridization rate and high number of single paralogue gene families may be a result of weak signals among diverged paralogues in both probe pools due to Tm's that approached the stringency of the washes. Alternately, some EST sequences may not be competitive; either in mixed pool probe synthesis by primed synthesis or in hybridization [43]. Alternately the MTP might not represent the entire soybean genome [5]. However, as judged by gel electrophoresis and re-sequencing the mean lengths of the ESTs were approximately 500 bp and most were from the 3' end of mRNAs. Therefore, some of the probes might have been gene specific [44]. The number of unique bands in the fingerprints of the MTP clones was 300,000 (each band represents about 4 kbp) [12]. The 3' UTR of most soybean ESTs is less than 500 bp [4]. Therefore, it is unlikely that the combination of; probes were gene specific probes that were too weak to be scored; and regions of the genome absent from the MTP would cause 35% of gene families to falsely appear to contain a single member.

Map locations were inferred for ~54% (108/201) of the EST paralogues to the soybean physical map (66 from FiS library and 42 from Gm-r1021 library). The other 12% hybridized to BAC clones that have been removed by manual editing from the physical map build 4. Further analysis of BAC clone fingerprints used in the MTP will place these ESTs on the physical map in future. The ESTs representing β-galactosidase, MAP kinase kinase alpha protein kinase, kinesin-like protein A, and calmodulin-stimulated calcium ATPase hybridized to the BAC clones of the soybean physical map that have been located on MLG C2 (a genetic map). However these EST/BAC clone combinations were not on the same contig, therefore not clustered. Three hundred thirty-seven BAC clones in 131 contigs represented the mapped ESTs. About 4% of the genome encompassed the selected defense-related genes. Genome sequence analysis of *Arabidopsis *showed that 11.5% of the genome is occupied by defense-related genes [40]. Therefore, the set of ESTs used may represent about one third of the soybean defense related genes. Further experiments should include the remaining defense-related genes in the soybean genome in order to improve physical mapping of defense related genes.

The results of this study located ESTs on linkage groups anchored by DNA markers. In September 2006 about 730 RFLP markers and 1,407 microsatellite markers were anchored to the genetic map [45]; whereas only 212 RFLPs (N. Young personal communication) and 404 microsatellite markers were sufficiently reliable to be anchored to the physical map [5,12]. G-browse shows markers anchored to EST paralogue hybridizing contigs. Comparison of marker locations with the consensus map can give a relative idea of the genetic locations and distributions of the particular gene family that the EST probe represented. The contig 1120 contains ESTs homologous to calcium binding protein isolog and calcium dependent protein kinase assigned to a MLG H and overlaps with QTL for resistance to corn ear worm. Many contigs were not assigned to LGs due to the lack of suitable anchored SSR markers. However about half of the contigs that contained paralogues of defense related genes mapped to locations that overlap with QTL for resistance to biotic factors. When resistance to abiotic stress was included, close to 80 % of contigs overlap either biotic or abiotic stress resistance QTL. Most contigs contain unique BAC end sequences and will be assigned to LGs during assembly of the whole genome shotgun sequence of soybean.

The biggest cluster of genes of related function was identified on contig 1751 that has not yet been mapped to a MLG. The ESTs that cluster on this contig include homologues of 4-coumarate:CoA ligase isoform 2 (AI442373), 7-O-methyltransferase (AI444115), β-galactosidase (AI441809), calmodulin (AI437703), calmodulin-like protein (AI442296), calmodulin-stimulated calcium ATPase (AI460618), casein kinase II beta chain (AI442731), CLV 1 receptor kinase (AI461073), epoxide hydrolase (AI438014), glycines cleavage system H protein precursor (AI437618), MAP kinase kinase alpha protein kinase (AI440721), proline-rich 14 KDA protein (AI443444), protein disulfide isomerase (AI437977), quinone oxidoreductase (AI437535), and threonine synthase (AI437902). In future studies, it will be interesting to know what QTL overlap with this contig.

At another unmapped location two ESTs with homology to 7-O-methyltransferase (AI444115) and *Medicago sativa *isoflavone-O-methyltransferase mRNA (BI245401) clustered together on contig 191. As a result this or other adjoining contigs may include genes important for isoflavone biosynthesis and the region may be involved in fungal growth/infection inhibition.

The distribution of the ESTs within the genome was interesting. Based on the hybridization of 201 ESTs (a limited number compared to the total soybean genes), many of the clustering ESTs were found in multiple positions in the physical map (build 3 and 4). For example, the EST BI347339, a homologue of *G. max *myo-inositol-1-phosphate synthase was found on two different contigs. Similarly, EST BI119573, a paralogue of *G. max *ascorbate peroxidase was found at 5 different locations on the physical map. Reasons for the multiple sites may be attributed to soybean's highly repetitive and duplicated genome or the higher copy number of these and other genes (Table 5). One of these genes is likely to be located on linkage group C2 where a peroxisomal ascorbate peroxidase (gi014240664) was found within a syntenic region in *M. truncatulata *(Dr. WD Beavis, personal communication) in a region underlying resistance to SDS.

Among the ESTs that we found in the unique gene family were homologous to known genes G-box binding factor, epoxide hydrolase, chalcone synthase, and phenyl alanine ammonia lyase 1 (PAL1). Southern hybridizations to genomic DNA with G-box factor probes found 5–7 copies in the genome ([46]; Table 5). There were five copies of epoxide hydrolase [47]. There were 8–9 copies of chalcone synthase genes [48,49] found at six loci. CHS1, CHS3, CHS4, dCHS1, were on a single BAC and CHS5 was 0.3 cM away on molecular linkage group (MLG) A2. CHS2 (A2), CHS6 (K), CHS7 (D1a) and CHS8 (B1) were all unlinked. There were 2 copies of PAL genes (48). In this study, we observed that there was only 1 copy for each of the above genes. This might suggest that the MTP does not represent the entire genome. However, equally likely explanations include that some gene families diverge rapidly; therefore, the stringency we used for the selected probe hybridization identified a single gene family member. For example the CHS gene family with 7 known members in nr was composed of two diverged clusters, type 1 and type 2 in Unigene.

Conversely an overabundance of hybridizing BACs was found in the analysis of the EST homologous to 4-coumarate CoA ligase 1. There were five BACs from different contigs. However, Southern hybridization and cDNA cloning inferred there were only 3 gene family members [50]. Therefore the MTP might be over-represented in some regions or some gene family members were overlooked in earlier studies. Each of the five BACs that hybridized were located in different contigs favoring the latter hypothesis. Further editing of the MTP is in progress [7] and two new MTPs have been developed to test such conclusions further.

Good correspondence was found among some ESTs homologous to known genes. Southern analysis performed on the calmodulin gene found four copies [51] coinciding with our finding of five copies. Nodulin 22 gene was also analyzed and was found to be located in 4–5 different locations in the genome [52] consistent with the five locations found on the MTP.

Our study found rather few (21; 10.4%) ESTs that belonged to three member gene families. Among these were ESTs homologous to ATP synthase, aspartate aminotransferase 1, and leghemoglobin. The gene number estimates coincided well with the reported Southern hybridization gene copy number estimates. ATP synthase was suggested to have 2–3 copies in the genome [53]. There were 1–2 copies of aspartate aminotransferase [54]. Two gene copies of leghemoglobin were inferred from Southerns [55]. The correspondence among BAC and Southern hybridizations with the 3 member gene families shows adequate genome representation by the MTP and may infer genes in three member gene families diverged more slowly than the other probes.

Arabidopsis, a model plant with a complete genome, was used to compare gene family sizes in soybean. A genome sequence analysis of Arabidopsis [40] found that 35% of the genome were unique genes (found only in one position in the genome). However, the genome duplications inferred for *A. thaliana *and *G.max *must have eliminated all unique genes, for soybean as recently as 4 MYA [2,4,5]. As a recent paleo-tetraploid, soybean was expected to contain no unique singleton genes (Table 3). In fact, about 35% of the genes selected were present in the gene families with one member suggesting rapid and genome wide divergence or gene loss in soybean. For gene families that contained two members, there were 12.5% in Arabidopsis compared to 25% in soybean, a clear effect of genome duplication. However, the 10.4% of genes in the three member gene families of soybean was similar in size to the 7% found in Arabidopsis. Again gene families in this class tended to be highly conserved. The gene families that contained four members occupied 4% of the Arabidopsis genome compared to 10.4% of the soybean genome. Again the effect of genome duplication in soybean was inferred. The five member gene families were approximately the same size in soybean (4.5%) and Arabidopsis (3.6%) suggesting rapid and genome wide divergence or gene loss in soybean. Finally, 37.4% of the Arabidopsis genome gene families had more than five members but only 15% for soybean.

The presence of twice the number of genes in the two and four member gene families in soybean compared to Arabidopsis may be due to the paleo-auto-tetraploid nature of the soybean genome [2-7]. The three member gene family was also slightly higher in soybean compared to Arabidopsis. However, the trend was reversed in 5 or greater member number gene families. The gene family size trends suggest their evolution is under strong selection. Comparable data were not available in 2006 for *Medicago truncatulata *or *Populus*. However from the rice (*Oryza sativa*) genome sequence [56] and tomato (*Lycopersicum esculentum*) [57] EST collection gene family size estimates were made. Rice had more unigenes than Arabidopsis or soybean but fewer 2 or 3–5 gene member families. Tomato had more than double the number of unigenes than Arabidopsis or soybean and was increased about only slightly for genes with 2 members, not to the degree inferred for soybean. Tomato gene families of 3 or more genes were only slightly less abundant than in Arabidopsis and in proportion (no bias against the 3 gene family members. These trends are consistent with the hypothesis that gene family size may be the sum of deletions during the genome shuffling and rearrangements occurring during the diploidization of the tetraploid genome. Further studies should examine gene-family size in soybean in relation to location on chromosomes as the genome sequence emerges and the physical map is completed [5].

## Supplementary Material

Additional file 1 Table of BACs identified in pool hybridizations of ESTs from the Gm-r1021 soybean cDNA library. The hybridized BACs are listed by common probe and Contig No. in ascending, numerical order. Click here for file

Additional file 2 Table of BAC hybridizations with ESTs from the FiS soybean cDNA library. The hybridized BACs are listed by common probe and Contig No. in ascending, numerical order Click here for file

Additional file 3 Table of ESTs found on major linkages groups of soybean in build 4. BACs that hybridized to ESTs sorted by linkage group to identify ESTs found on the different MLGs. Click here for file

Additional file 4 Locations inferred in the build 3 version of the physical map of BAC clones hybridized to subtracted ESTs showing contig number and linkage group if determined. BACs that hybridized to ESTs in build 3 sorted by linkage group to identify ESTs found on the different MLGs. Click here for file
