# Supplementary material for: Development of a pooled probe method for locating small gene families in a physical map of soybean using stress related paralogues and a BAC minimum tile path
Source: Plant Methods. 2006 Dec 8;2:20. doi: 10.1186/1746-4811-2-20 (PMC1716159; doi:10.1186/1746-4811-2-20)
Supplement: Additional file 1 — Table of BACs identified in pool hybridizations of ESTs from the Gm-r1021 soybean cDNA library. The hybridized BACs are listed by common probe and Contig No. in ascending, numerical order. [file 1746-4811-2-20-S1.doc]

| Additional file 1: Table of BACs identified in pool hybridizations of ESTs from the Gm-r1021 soybean cDNA library. The hybridized BACs are listed by common probe and Contig No. in ascending, numerical order. | | | | | |
| --- | --- | --- | --- | --- | --- |
|  | |  |  |  |  |
|  |  |  |  |  |  |
| **BAC clone** | **EST (SIU ID)** | **GenBank Accession No.** | **EST Homolog** | **Contig No.** | **MLG** |
| H13E17 | Gm-r1021-10C13 | AI494845 | Lipase isolog | ** | ** |
| H49O16 | Gm-r1021-10C13 | AI494845 | Lipase isolog | ** | ** |
| H30L05 | Gm-r1021-10L17 | AI495603 | Repetitive proline-rich cell wall protein 2 precursor | ** | ** |
| H29B13 | Gm-r1021-11A13 | AI494734 | Histidine kinase A | ** | ** |
| H38F19 | Gm-r1021-11A13 | AI494734 | Histidine kinase A | ** | ** |
| H41D18 | Gm-r1021-1A17 | AI416624 | 13-Lipoxygenase | ** | ** |
| B36K23 | Gm-r1021-1B10 | AI444067 | Ca2+ ATPase | ** | ** |
| B42H09 | Gm-r1021-1B10 | AI444067 | Ca2+ ATPase | ** | ** |
| B12L12 | Gm-r1021-1B18 | AI443454 | Putative water channel protein | ** | ** |
| B38M08 | Gm-r1021-1B18 | AI443454 | Putative water channel protein | ** | ** |
| H36G12 | Gm-r1021-1B18 | AI443454 | Putative water channel protein | ** | ** |
| H67E16 | Gm-r1021-1B18 | AI443454 | Putative water channel protein | ** | ** |
| B38M08 | Gm-r1021-1C23 | AI416617 | Spermidine synthase 2 | ** | ** |
| H43J22 | Gm-r1021-1C23 | AI416617 | Spermidine synthase 2 | ** | ** |
| B38M08 | Gm-r1021-1D08 | AI443444 | Proline-rich 14 KDA protein | ** | ** |
| B39I18 | Gm-r1021-1D08 | AI443444 | Proline-rich 14 KDA protein | ** | ** |
| H04A01 | Gm-r1021-1D08 | AI443444 | Proline-rich 14 KDA protein | ** | ** |
| H36G12 | Gm-r1021-1D08 | AI443444 | Proline-rich 14 KDA protein | ** | ** |
| H68D20 | Gm-r1021-1D08 | AI443444 | Proline-rich 14 KDA protein | ** | ** |
| H36G12 | Gm-r1021-1D12 | AI443042 | Chalcone synthase 7 | ** | ** |
| B38M08 | Gm-r1021-1D14 | AI443060 | Chitinase homolog LP6 | ** | ** |
| H43J22 | Gm-r1021-1D14 | AI443060 | Chitinase homolog LP6 | ** | ** |
| H47M11 | Gm-r1021-1E16 | AI442505 | Sucrose transporter | ** | ** |
| B35N24 | Gm-r1021-1E17 | AI416641 | Succinyl-CoA ligase | ** | ** |
| B38M08 | Gm-r1021-1J05 | AI444097 | Acanthamoeba polyubiquitin gene | ** | ** |
| H36G12 | Gm-r1021-1J15 | AI443817 | Phosphoribosylformylglycinamidine cyclo-ligase precursor | ** | ** |
| Table 2 continued | | | | | |
| H43J22 | Gm-r1021-1J15 | AI443817 | Phosphoribosylformylglycinamidine cyclo-ligase precursor | ** | ** |
| B12L12 | Gm-r1021-1K04 | AI442439 | 5'-Bisphosphate nucleotidase | ** | ** |
| B38M08 | Gm-r1021-1K04 | AI442439 | 5'-Bisphosphate nucleotidase | ** | ** |
| B12L12 | Gm-r1021-1K08 | AI442507 | Cu2+ transporting ATPase homolog | ** | ** |
| B38M08 | Gm-R1021-1K08 | AI442507 | Cu2+ transporting ATPase homolog | ** | ** |
| H11O01 | Gm-R1021-1K08 | AI442507 | Cu2+ transporting ATPase homolog | ** | ** |
| H68D20 | Gm-R1021-1K08 | AI442507 | Cu2+ transporting ATPase homolog | ** | ** |
| B38M08 | Gm-R1021-1K24 | AI443974 | TMV resistance protein homolog. | ** | ** |
| B54E14 | Gm-R1021-1K24 | AI443974 | TMV resistance protein homolog. | ** | ** |
| H43J22 | Gm-R1021-1K24 | AI443974 | TMV resistance protein homolog. | ** | ** |
| H68D20 | Gm-R1021-1K24 | AI443974 | TMV resistance protein homolog. | ** | ** |
| H68D20 | Gm-R1021-1L01 | AI444109 | Polyubiquitin | ** | ** |
| B16E09 | Gm-r1021-1L24 | AI442570 | Beta-ureidopropionase | ** | ** |
| B19G14 | Gm-r1021-1L24 | AI442570 | Beta-ureidopropionase | ** | ** |
| B38M08 | Gm-r1021-1L24 | AI442570 | Beta-ureidopropionase | ** | ** |
| H43J22 | Gm-r1021-1L24 | AI442570 | Beta-ureidopropionase | ** | ** |
| H68D20 | Gm-r1021-1L24 | AI442570 | Beta-ureidopropionase | ** | ** |
| H74M21 | Gm-r1021-1L24 | AI442570 | Beta-ureidopropionase | ** | ** |
| B39I18 | Gm-r1021-1M21 | AI442373 | 4-Coumarate:CoA ligase isoform 2 | ** | ** |
| H36G12 | Gm-r1021-1M21 | AI442373 | 4-Coumarate:CoA ligase isoform 2 | ** | ** |
| H01A23 | Gm-R1021-1N23 | AI442586 | Phosphoribosylamidoimidazole-succinocarboxamide synthase precursor | ** | ** |
| B38M08 | Gm-r1021-1P11 | AI444099 | 3-Isopropylmalate dehydratase, leud subunit. | ** | ** |
| H03E20 | Gm-r1021-1P11 | AI444099 | 3-Isopropylmalate dehydratase, leud subunit. | ** | ** |
| B38M08 | Gm-r1021-2A10 | AI437501 | 6-Phosphogluconate dehydrogenase | ** | ** |
| H36G12 | Gm-r1021-2A10 | AI437501 | 6-Phosphogluconate dehydrogenase | ** | ** |
| H43J22 | Gm-r1021-2A10 | AI437501 | 6-Phosphogluconate dehydrogenase | ** | ** |
| B38M08 | Gm-r1021-2B16 | AI437902 | Threonine synthase | ** | ** |
| H04A01 | Gm-r1021-2B16 | AI437902 | Threonine synthase | ** | ** |
| H25F14 | Gm-r1021-2B16 | AI437902 | Threonine synthase | ** | ** |
| Table 2 continued | | | | | |
| H43J22 | Gm-r1021-2B16 | AI437902 | Threonine synthase | ** | ** |
| H68D20 | Gm-r1021-2B16 | AI437902 | Threonine synthase | ** | ** |
| H12B04 | Gm-r1021-2C03 | AI444115 | 7-O-methyltransferase | ** | ** |
| H25F14 | Gm-r1021-2C03 | AI444115 | 7-O-methyltransferase | ** | ** |
| B38B14 | Gm-r1021-2C05 | AI442530 | Amidophosphoribosyltransferase | ** | ** |
| B38M08 | Gm-r1021-2C05 | AI442530 | Amidophosphoribosyltransferase | ** | ** |
| H36G12 | Gm-r1021-2C05 | AI442530 | Amidophosphoribosyltransferase | ** | ** |
| B15I12 | Gm-r1021-2C08 | AI437977 | Protein disulfide isomerase | ** | ** |
| H36G12 | Gm-r1021-2C08 | AI437977 | Protein disulfide isomerase | ** | ** |
| H57E20 | Gm-r1021-2C08 | AI437977 | Protein disulfide isomerase | ** | ** |
| B38M08 | Gm-r1021-2C14 | AI437618 | Glycine cleavage system H protein precursor | ** | ** |
| B44K21 | Gm-r1021-2C20 | AI437968 | Cytoplasmic aldolase | ** | ** |
| H49L04 | Gm-r1021-2C20 | AI437968 | Cytoplasmic aldolase | ** | ** |
| H36G12 | Gm-r1021-2E06 | AI437535 | Quinone oxidoreductase | ** | ** |
| H57E20 | Gm-r1021-2E06 | AI437535 | Quinone oxidoreductase | ** | ** |
| H68D20 | Gm-r1021-2E06 | AI437535 | Quinone oxidoreductase | ** | ** |
| B38M08 | Gm-r1021-2E16 | AI437503 | Indole-3-acetate beta-glucosyltransferase isolog | ** | ** |
| H43J22 | Gm-r1021-2E16 | AI437503 | Indole-3-acetate beta-glucosyltransferase isolog | ** | ** |
| H68D20 | Gm-r1021-2E16 | AI437503 | Indole-3-acetate beta-glucosyltransferase isolog | ** | ** |
| B24K04 | Gm-r1021-2E24 | AI437544 | Putative membrane intrinsic protein | ** | ** |
| H17I22 | Gm-r1021-2E24 | AI437544 | Putative membrane intrinsic protein | ** | ** |
| B38M08 | Gm-r1021-2F06 | AI437703 | Calmodulin | ** | ** |
| H47F05 | Gm-r1021-2F06 | AI437703 | Calmodulin | ** | ** |
| H72C10 | Gm-r1021-2F06 | AI437703 | Calmodulin | ** | ** |
| B38M08 | Gm-r1021-2H06 | AI437704 | Cellulose synthase | ** | ** |
| B53N17 | Gm-r1021-2H06 | AI437704 | Cellulose synthase | ** | ** |
| B33A16 | Gm-r1021-2H22 | AI437753 | Carboxyl-terminal proteinase homolog | ** | ** |
| H62J13 | Gm-r1021-2I02 | AI437504 | Phenylalanyltrna synthetase | ****** | ** |
| B14I07 | Gm-r1021-2I06 | AI437999 | ADR12 protein | ** | ** |
| H14O24 | Gm-r1021-2I23 | AI437632 | 4-Coumarate CoA ligase 1 | ** | ** |
| H43J22 | Gm-r1021-2I23 | AI437632 | 4-Coumarate CoA ligase 1 | ** | ** |
| Table 2 continued | | | | | |
| H52B14 | Gm-r1021-2I23 | AI437632 | 4-Coumarate CoA ligase 1 | ** | ** |
| H68D20 | Gm-r1021-2I23 | AI437632 | 4-Coumarate CoA ligase 1 | ** | ** |
| B12L12 | Gm-r1021-2J02 | AI437799 | Anionic peroxidase precursor | ** | ** |
| B38M08 | Gm-r1021-2J02 | AI437799 | Anionic peroxidase precursor | ** | ** |
| B07K01 | Gm-r1021-2J05 | AI437948 | MAP3K delta 1 protein kinase | ** | ** |
| B38M08 | Gm-r1021-2J05 | AI437948 | MAP3K delta 1 protein kinase | ** | ** |
| H43J22 | Gm-r1021-2J05 | AI437948 | MAP3K delta 1 protein kinase | ** | ** |
| H68D20 | Gm-r1021-2J05 | AI437948 | MAP3K delta 1 protein kinase | ** | ** |
| B35K11 | Gm-r1021-2K11 | AI437531 | Fructokinase | ** | ** |
| B38M08 | Gm-r1021-2K11 | AI437531 | Fructokinase | ** | ** |
| H43J22 | Gm-r1021-2K11 | AI437531 | Fructokinase | ** | ** |
| H68D20 | Gm-r1021-2K11 | AI437531 | Fructokinase | ** | ** |
| B38M08 | Gm-r1021-2K13 | AI437572 | Endochitinase | ** | ** |
| B38M08 | Gm-r1021-2L12 | AI437773 | Cytochrome P450 | ** | ** |
| B54I06 | Gm-r1021-2L12 | AI437773 | Cytochrome P450 | ** | ** |
| H14O24 | Gm-r1021-2L12 | AI437773 | Cytochrome P450 | ** | ** |
| B38M08 | Gm-r1021-2L18 | AI437885 | G-box binding factor | ** | ** |
| B38M08 | Gm-r1021-2N04 | AI437818 | Fumarylacetoacetase | ** | ** |
| B24E01 | Gm-r1021-2O02 | AI437613 | Thioredoxin | ** | ** |
| B38M08 | Gm-r1021-2O02 | AI437613 | Thioredoxin | ** | ** |
| H45F16 | Gm-r1021-2P15 | AI437780 | Leucine-rich repeat/receptor protein kinase precursor | ** | ** |
| B12L10 | Gm-r1021-3A11 | AI437905 | Sulfate adenylyltransferase | ** | ** |
| H26F07 | Gm-r1021-3A11 | AI437905 | Sulfate adenylyltransferase | ** | ** |
| H26F07 | Gm-r1021-3A23 | AI437820 | Vascular actin single-stranded DNA-binding factor 2 P44 component | ** | ** |
| B38M08 | Gm-r1021-3B08 | AI442231 | Catalase | ** | ** |
| B38M08 | Gm-r1021-3B19 | AI443896 | Aspartate aminotransferase 1 | ** | ** |
| H56J20 | Gm-r1021-3B19 | AI443896 | Aspartate aminotransferase 1 | ** | ** |
| B38M08 | Gm-r1021-3B20 | AI441818 | Caffeic acid O-methyltransferase | ** | ** |
| B38M08 | Gm-r1021-3D02 | AI443293 | Methionine synthase | ** | ** |
| H17M13 | Gm-r1021-3D02 | AI443293 | Methionine synthase | ** | ** |
| Table 2 continued | | | | | |
| B38M08 | Gm-r1021-3D10 | AI442210 | Aspartokinase | ** | ** |
| H32G20 | Gm-r1021-3D10 | AI442210 | Aspartokinase | ****** | ** |
| H68D20 | Gm-r1021-3E04 | AI442612 | Enolase | ** | ** |
| B38M08 | Gm-r1021-3G21 | AI443889 | Glycine rich protein | ** | ** |
| H11K13 | Gm-r1021-3H12 | AI441809 | Beta-galactosidase | ** | ** |
| H25F14 | Gm-r1021-3H12 | AI441809 | Beta-galactosidase | ** | ** |
| H36G12 | Gm-r1021-3H12 | AI441809 | Beta-galactosidase | ** | ** |
| H43J22 | Gm-r1021-3H12 | AI441809 | Beta-galactosidase | ** | ** |
| H57E20 | Gm-r1021-3H12 | AI441809 | Beta-galactosidase | ** | ** |
| H57F07 | Gm-r1021-3H12 | AI441809 | Beta-galactosidase | ** | ** |
| H68D20 | Gm-r1021-3H12 | AI441809 | Beta-galactosidase | ** | ** |
| H52I12 | Gm-r1021-3H22 | AI443248 | Calnexin | ** | ** |
| H13K12 | Gm-r1021-3I05 | AI443638 | Ketol-acid reductoisomerase precursor | ** | ** |
| H46M19 | Gm-r1021-3I07 | AI442627 | Aminotransferase | ** | ** |
| B35G05 | Gm-r1021-3I24 | AI442632 | Tonoplast intrinsic protein, root-specific RB7-5A | ** | ** |
| B48B24 | Gm-r1021-3I24 | AI442632 | Tonoplast intrinsic protein, root-specific RB7-5A | ** | ** |
| H68D20 | Gm-r1021-3K01 | AI443648 | Homeoboxleucine zipper protein ATHB6 | ** | ** |
| H11B10 | Gm-r1021-3K03 | AI442658 | Calcium binding protein isolog | ** | ** |
| H68D20 | Gm-r1021-3K03 | AI442658 | Calcium binding protein isolog | ** | ** |
| B38M08 | Gm-r1021-3N22 | AI442245 | Vacuolar ATP synthase subunit C | ** | ** |
| B38M08 | Gm-r1021-3O24 | AI443886 | Malonyl CoA-acyl carrier protein transacylase | ** | ** |
| H07J04 | Gm-r1021-3O24 | AI443886 | Malonyl CoA-acyl carrier protein transacylase | ** | ** |
| H43J22 | Gm-r1021-3O24 | AI443886 | Malonyl CoA-acyl carrier protein transacylase | ** | ** |
| H68D20 | Gm-r1021-3O24 | AI443886 | Malonyl CoA-acyl carrier protein transacylase | ** | ** |
| H68D20 | Gm-r1021-3P18 | AI443229 | Carbamoyl phosphate synthetase large chain | ** | ** |
| H11B04 | Gm-r1021-4A05 | AI443299 | ATP-citrate | ** | ** |
| H24D16 | Gm-r1021-4A05 | AI443299 | ATP-citrate | ** | ** |
| H24G14 | Gm-r1021-4B19 | AI440861 | 3-Deoxy-D-arabino-heptulosonate 7-phosphate synthase | ** | ** |
| H67E21 | Gm-R1021-4C18 | AI443166 | Pectate lyase | ** | ** |
| H01A23 | Gm-r1021-4D05 | AI440931 | 1-Aminocyclopropane-1-carboxylate oxidase | ** | ** |
| H39F10 | Gm-r1021-4D05 | AI440931 | 1-Aminocyclopropane-1-carboxylate oxidase | ** | ** |
| Table 2 continued | | | | | |
| H61M06 | Gm-r1021-4D05 | AI440931 | 1-Aminocyclopropane-1-carboxylate oxidase | ** | ** |
| H68D20 | Gm-r1021-4O16 | AI441234 | Abscisic stress ripening protein 1 | ** | ** |
| B48B24 | Gm-r1021-4O19 | AI441937 | Chalcone synthase | ** | ** |
| H52I12 | Gm-r1021-4P01 | AI440894 | Serine/threonine kinase | ** | ** |
| H52I12 | Gm-r1021-5B03 | AI440630 | Putative potassium transporter ATKT2P | ** | ** |
| H68D20 | Gm-r1021-5H08 | AI441087 | Protein kinase | ** | ** |
| B35G21 | Gm-r1021-5I21 | AI441043 | Vacuolar ATP synthase catalytic subunit A | ** | ** |
| B27M18 | Gm-r1021-5J01 | AI441021 | Calciumdependent protein kinase | ** | ** |
| B35K11 | Gm-r1021-5J01 | AI441021 | Calciumdependent protein kinase | ** | ** |
| H11B10 | Gm-r1021-5J01 | AI441021 | Calciumdependent protein kinase | ** | ** |
| H60L07 | Gm-r1021-5J01 | AI441021 | Calciumdependent protein kinase | ** | ** |
| H68D20 | Gm-r1021-5J01 | AI441021 | Calciumdependent protein kinase | ** | ** |
| B15I12 | Gm-r1021-5J22 | AI442296 | Calmodulinlike protein | ** | ** |
| H25F14 | Gm-r1021-5J22 | AI442296 | Calmodulinlike protein | ** | ** |
| H36G12 | Gm-r1021-5J22 | AI442296 | Calmodulinlike protein | ** | ** |
| H57E20 | Gm-r1021-5J22 | AI442296 | Calmodulinlike protein | ** | ** |
| B38M08 | Gm-r1021-5K20 | AI440721 | MAP kinase kinase alpha protein kinase | ** | ** |
| B39I18 | Gm-r1021-5K20 | AI440721 | MAP kinase kinase alpha protein kinase | ** | ** |
| H04A01 | Gm-r1021-5K20 | AI440721 | MAP kinase kinase alpha protein kinase | ** | ** |
| H25F14 | Gm-r1021-5K20 | AI440721 | MAP kinase kinase alpha protein kinase | ** | ** |
| H36G12 | Gm-r1021-5K20 | AI440721 | MAP kinase kinase alpha protein kinase | ** | ** |
| H43J22 | Gm-r1021-5K20 | AI440721 | MAP kinase kinase alpha protein kinase | ** | ** |
| H68D20 | Gm-r1021-5K20 | AI440721 | MAP kinase kinase alpha protein kinase | ** | ** |
| B38I03 | Gm-r1021-6A22 | AI441632 | Salt-tolerance protein | ** | ** |
| B38M08 | Gm-r1021-6D10 | AI460671 | Kinesinlike protein A | ** | ** |
| B39I18 | Gm-r1021-6D10 | AI460671 | Kinesinlike protein A | ** | ** |
| H04A01 | Gm-r1021-6D10 | AI460671 | Kinesinlike protein A | ** | ** |
| H25F14 | Gm-r1021-6D10 | AI460671 | Kinesinlike protein A | ** | ** |
| H36G12 | Gm-r1021-6D10 | AI460671 | Kinesinlike protein A | ** | ** |
| H48E11 | Gm-r1021-6D10 | AI460671 | Kinesinlike protein A | ** | ** |
| H58J10 | Gm-r1021-6D10 | AI460671 | Kinesinlike protein A | ** | ** |
| Table 2 continued | | | | | |
| H30B10 | Gm-r1021-6E18 | AI440615 | Extracellular dermal glycoprotein precursor | ** | ** |
| B33C24 | Gm-r1021-6F17 | AI460654 | Stress related protein PVSRP | ** | ** |
| B38M08 | Gm-r1021-6F17 | AI460654 | Stress related protein PVSRP | ** | ** |
| H52I12 | Gm-r1021-6H24 | AI461080 | Ethylene-forming enzyme | ** | ** |
| B15I12 | Gm-r1021-6J19 | AI460618 | Calmodulin-stimulated calcium ATPase | ** | ** |
| B39I18 | Gm-R1021-6J19 | AI460618 | Calmodulin-stimulated calcium ATPase | ** | ** |
| H25F14 | Gm-R1021-6J19 | AI460618 | Calmodulin-stimulated calcium ATPase | ** | ** |
| H36G12 | Gm-R1021-6J19 | AI460618 | Calmodulin-stimulated calcium ATPase | ** | ** |
| B39I18 | Gm-r1021-6J24 | AI522819 | Waterstress induced tonoplast intrinsic protein | ** | ** |
| H25F14 | Gm-r1021-6J24 | AI522819 | Waterstress induced tonoplast intrinsic protein | ** | ** |
| H36G12 | Gm-r1021-6J24 | AI522819 | Waterstress induced tonoplast intrinsic protein | ** | ** |
| B54I06 | Gm-r1021-6M15 | AI440582 | 4-Coumarate CoA ligase | ** | ** |
| H06E01 | Gm-r1021-6M15 | AI440582 | 4-Coumarate CoA ligase | ** | ** |
| B34L17 | Gm-r1021-6M24 | AI461071 | Beta tubulin | ** | ** |
| B38E15 | Gm-r1021-6M24 | AI461071 | Beta tubulin | ** | ** |
| B38M08 | Gm-r1021-6M24 | AI461071 | Beta tubulin | ** | ** |
| H38F03 | Gm-r1021-6M24 | AI461071 | Beta tubulin | ** | ** |
| H50I21 | Gm-r1021-6M24 | AI461071 | Beta tubulin | ** | ** |
| B38M08 | Gm-r1021-7D02 | AI442731 | Casein kinase II beta chain | ** | ** |
| H12B17 | Gm-r1021-7D02 | AI442731 | Casein kinase II beta chain | ** | ** |
| B38M08 | Gm-r1021-7D20 | AI441758 | Flavonol synthase | ** | ** |
| H30M22 | Gm-R1021-7E09 | AI522876 | DAG protein precursor | ****** | ** |
| B46J19 | Gm-r1021-7F01 | AI460590 | ACC-oxidase | ** | ** |
| H66G11 | Gm-r1021-7G21 | AI461215 | Similarity to AMP activated protein kinase beta | ** | ** |
| B51M12 | Gm-r1021-7I16 | AI460495 | Vacuolar ATP synthase subunit B isoform 1 | ** | ** |
| H30J11 | Gm-r1021-7I16 | AI460495 | Vacuolar ATP synthase subunit B isoform 1 | ** | ** |
| B35H04 | Gm-r1021-7I21 | AI522838 | NAD(P)H dependent 6'deoxychalcone synthase | ** | ** |
| H35J06 | Gm-r1021-8G05 | AI507864 | 7-O-methyltransferase | ** | ** |
| B02G20 | Gm-r1021-6J24 | AI522819 | Waterstress induced tonoplast intrinsic protein | ctg1079 | Queue |
| H35I01 | Gm-r1021-3K03 | AI442658 | Calcium binding protein isolog | ctg1120 | H |
| H35I01 | Gm-r1021-5J01 | AI441021 | Calciumdependent protein kinase | ctg1120 | H |
| Table 2 continued | | | | | |
| H31J02 | Gm-r1021-2B16 | AI437902 | Threonine synthase | ctg1443 | Queue |
| H31J02 | Gm-r1021-2E06 | AI437535 | Quinone oxidoreductase | ctg1443 | Queue |
| H31J02 | Gm-r1021-3H12 | AI441809 | Beta-galactosidase | ctg1443 | Queue |
| H31J02 | Gm-r1021-5K20 | AI440721 | MAP kinase kinase alpha protein kinase | ctg1443 | Queue |
| H31J02 | Gm-r1021-6D10 | AI460671 | Kinesinlike protein A | ctg1443 | Queue |
| H39A16 | Gm-r1021-1D08 | AI443444 | Proline-rich 14 KDA protein | ctg1751 | Queue |
| H39A16 | Gm-r1021-1M21 | AI442373 | 4-Coumarate:CoA ligase isoform 2 | ctg1751 | Queue |
| H39A16 | Gm-r1021-2B16 | AI437902 | Threonine synthase | ctg1751 | Queue |
| H39A16 | Gm-r1021-2C03 | AI444115 | 7-O-methyltransferase | ctg1751 | Queue |
| H39A16 | Gm-r1021-2C08 | AI437977 | Protein disulfide isomerase | ctg1751 | Queue |
| H39A16 | Gm-r1021-2C14 | AI437618 | Glycine cleavage system H protein precursor | ctg1751 | Queue |
| H39A16 | Gm-r1021-2E06 | AI437535 | Quinone oxidoreductase | ctg1751 | Queue |
| H39A16 | Gm-r1021-2F06 | AI437703 | Calmodulin | ctg1751 | Queue |
| H39A16 | Gm-r1021-2F09 | AI438014 | Epoxide hydrolase | ctg1751 | Queue |
| H39A16 | Gm-r1021-3H12 | AI441809 | Beta-galactosidase | ctg1751 | Queue |
| H39A16 | Gm-r1021-5J22 | AI442296 | Calmodulinlike protein | ctg1751 | Queue |
| H39A16 | Gm-r1021-5K20 | AI440721 | MAP kinase kinase alpha protein kinase | ctg1751 | Queue |
| H39A16 | Gm-r1021-6D13 | AI461073 | CLV1 receptor kinase | ctg1751 | Queue |
| H39A16 | Gm-R1021-6J19 | AI460618 | Calmodulin-stimulated calcium ATPase | ctg1751 | Queue |
| H39A16 | Gm-r1021-7D02 | AI442731 | Casein kinase II beta chain | ctg1751 | Queue |
| H45O20 | Gm-r1021-2C03 | AI444115 | 7-O-methyltransferase | ctg191 | Queue |
| H19B13 | Gm-r1021-4D05 | AI440931 | 1-Aminocyclopropane-1-carboxylate oxidase | ctg2108 | K |
| B23M01 | Gm-r1021-3H22 | AI443248 | Calnexin | ctg2657 | Queue |
| B23M01 | Gm-r1021-4P01 | AI440894 | Serine/threonine kinase | ctg2657 | Queue |
| B23M01 | Gm-r1021-5B03 | AI440630 | Putative potassium transporter ATKT2P | ctg2657 | Queue |
| B23M01 | Gm-r1021-6H24 | AI461080 | Ethylene-forming enzyme | ctg2657 | Queue |
| B24C11 | Gm-r1021-3B19 | AI443896 | Aspartate aminotransferase 1 | ctg2704 | A2 & C2 |
| H18A04 | Gm-r1021-2E16 | AI437503 | Indole-3-acetate beta-glucosyltransferase isolog | ctg2892 | Queue |
| H63O23 | Gm-r1021-6D10 | AI460671 | Kinesinlike protein A | ctg2988 | Queue |
| H22H05 | Gm-r1021-3H12 | AI441809 | Beta-galactosidase | ctg3036 | C2 |
| H22H05 | Gm-r1021-6D10 | AI460671 | Kinesinlike protein A | ctg3036 | C2 |
| Table 2 continued | | | | | |
| B53F09 | Gm-r1021-2C08 | AI437977 | Protein disulfide isomerase | ctg3256 | Queue |
| B40B12 | Gm-r1021-6D10 | AI460671 | Kinesinlike protein A | ctg3303 | Queue |
| H42M11 | Gm-r1021-2H06 | AI437704 | Cellulose synthase | ctg3333 | Queue |
| B38E05 | Gm-r1021-5I21 | AI441043 | Vacuolar ATP synthase catalytic subunit A | ctg3520 | Queue |
| B50A20 | Gm-r1021-2E16 | AI437503 | Indole-3-acetate beta-glucosyltransferase isolog | ctg3687 | Queue |
| H20A11 | Gm-r1021-4B19 | AI440861 | 3-Deoxy-D-arabino-heptulosonate 7-phosphate synthase | ctg542 | Queue |
| H76D11 | Gm-R1021-4C18 | AI443166 | Pectate lyase | ctg558 | Queue |
| H76D11 | Gm-r1021-5H08 | AI441087 | Protein kinase | ctg558 | Queue |
| H39D15 | Gm-r1021-3H12 | AI441809 | Beta-galactosidase | ctg61 | Queue |
| H24J01 | Gm-r1021-1P11 | AI444099 | 3-Isopropylmalate dehydratase, leud subunit. | ctg641 | J |
| H41O12 | Gm-r1021-10H24 | AI495635 | Casein kinase II, alpha chain | ctg646 | Queue |
| H66A06 | Gm-r1021-5J22 | AI442296 | Calmodulinlike protein | ctg8120 | Queue |
| H66A06 | Gm-R1021-6J19 | AI460618 | Calmodulin-stimulated calcium ATPase | ctg8120 | Queue |
| H46H10 | Gm-r1021-2L12 | AI437773 | Cytochrome P450 | ctg8188 | D1AQ |
| B46F09 | Gm-r1021-6D10 | AI460671 | Kinesinlike protein A | ctg858 | Queue |
| H45M23 | Gm-r1021-6D10 | AI460671 | Kinesinlike protein A | ctg858 | Queue |
| H45M23 | Gm-r1021-6J24 | AI522819 | Waterstress induced tonoplast intrinsic protein | ctg858 | Queue |
| H13E14 | Gm-r1021-3N22 | AI442245 | Vacuolar ATP synthase subunit C | ctg9034 | Queue |
| H39D19 | Gm-r1021-3K03 | AI442658 | Calcium binding protein isolog | ctg9048 | Queue |
| H06B23 | Gm-r1021-2F03 | AI437497 | Calretulin | ctg9056 | I |
| B36N10 | Gm-r1021-6M24 | AI461071 | Beta tubulin | ctg9161 | B1 |
| B31L10 | Gm-r1021-3I07 | AI442627 | Aminotransferase | ctg9201 | Queue |
| B51L05 | Gm-r1021-5J01 | AI441021 | Calciumdependent protein kinase | ctg9210 | O |
| H58E12 | Gm-r1021-2E06 | AI437535 | Quinone oxidoreductase | ctg9240 | Queue |
| H58E12 | Gm-r1021-3H12 | AI441809 | Beta-galactosidase | ctg9240 | Queue |
| H40B02 | Gm-r1021-1B10 | AI444067 | Ca2+ ATPase | ctg9271 | Queue |
| H77N20 | Gm-r1021-2C08 | AI437977 | Protein disulfide isomerase | ctg9292 | D1AQ |
| H35G05 | Gm-r1021-5J01 | AI441021 | Calciumdependent protein kinase | ctg9310 | Queue |
| B14M21 | Gm-r1021-2F06 | AI437703 | Calmodulin | ctg9320 | Queue |
| B14M21 | Gm-r1021-7D02 | AI442731 | Casein kinase II beta chain | ctg9320 | Queue |
| B14M21 | Gm-r1021-7D20 | AI441758 | Flavonol synthase | ctg9320 | Queue |
| Table 2 continued | | | | | |
| H41O05 | Gm-r1021-3H12 | AI441809 | Beta-galactosidase | ctg9340 | Queue |
| H33O15 | Gm-r1021-2H06 | AI437704 | Cellulose synthase | ctg9349 | E |
| H77P02 | Gm-r1021-7D20 | AI441758 | Flavonol synthase | ctg9354 | G |
| H57O09 | Gm-r1021-5K20 | AI440721 | MAP kinase kinase alpha protein kinase | ctg9357 | C2 |
| H57O09 | Gm-R1021-6J19 | AI460618 | Calmodulin-stimulated calcium ATPase | ctg9357 | C2 |
| H44P15 | Gm-r1021-2I23 | AI437632 | 4-Coumarate CoA ligase 1 | ctg9361 | D1BW |
| B07L16 | Gm-r1021-5J22 | AI442296 | Calmodulinlike protein | ctg9376 | Queue |
| B07L16 | Gm-r1021-6J19 | AI460618 | Calmodulin-stimulated calcium ATPase | ctg9376 | Queue |

**Data not available
