# Supplementary material for: Development of a pooled probe method for locating small gene families in a physical map of soybean using stress related paralogues and a BAC minimum tile path
Source: Plant Methods. 2006 Dec 8;2:20. doi: 10.1186/1746-4811-2-20 (PMC1716159; doi:10.1186/1746-4811-2-20)
Supplement: Additional file 2 — Table of BAC hybridizations with ESTs from the FiS soybean cDNA library. The hybridized BACs are listed by common probe and Contig No. in ascending, numerical order [file 1746-4811-2-20-S2.doc]

| Additional file 2: Table of BAC hybridizations with ESTs from the FiS soybean cDNA library. The hybridized BACs are listed by common probe and Contig No. in ascending, numerical order. | | | | | |
| --- | --- | --- | --- | --- | --- |
|  | |  |  |  |  |
| **BAC clone ID** | **EST(SIU ID)** | **GenBank Accession No.** | **EST homolog** | **Contig No.** | **MLG** |
| B01B05 | FiS1i19 | BI273655 | *Glycine max* ascorbate peroxidase mRNA, complete cds. | ** | ** |
| B03H05 | FiS1D1 | BI245402 | *Glycine max* peroxidase precursor (GMIPER1) mRNA, complete cds. | ** | ** |
| B03K04 | FiS1B2 | BI119569 | EST | ** | ** |
| B03K04 | FiS2B2D11 | BM499239 | *Nicotiana attenuata* pathogen-inducible alpha-dioxygenase (PIOX_NICAT) mRNA, complete cds. | ** | ** |
| B03M12 | Fi37A24 | BI347331 | EST | ** | ** |
| B04A07 | Fi37A24 | BI347331 | EST | ** | ** |
| B04A07 | FiS1i6 | BI273669 | *Glycine max* Williams 82 lipoxygenase mRNA, complete cds. | ** | ** |
| B14I07 | Fi39D23 | BI347332 | *Glycine max* ADR12 mRNA | ** | ** |
| B14I07 | FiS1A16 | BI119554 | *Glycine max* ADR12 mRNA | ** | ** |
| B14I07 | FiS1G16 | BI273651 | *Glycine max* PAL1 gene for phenylalanine ammonia lyase (EC 4.3.1.5). | ** | ** |
| B14I07 | FiS1i3 | BI273639 | EST | ** | ** |
| B14O15 | FiS1A3 | BI273654 | EST | ** | ** |
| B16B01 | FiS1i7 | BI273640 | EST | ** | ** |
| B17D02 | FiS1M6 | BI245393 | EST | ** | ** |
| B19N19 | FiS2B2D11 | BM499239 | *Nicotiana attenuata* pathogen-inducible alpha-dioxygenase (PIOX_NICAT) mRNA, complete cds. | ** | ** |
| B23C13 | Fi36H18 | BI347330 | *Arabidopsis thaliana* putative elongation factor 1B alpha-subunit | ** | ** |
| B32C13 | Fi37A24 | BI347331 | EST | ** | ** |
| B32P09 | FiS1A17 | BI119550 | *Glycine max* actin (Soy57) gene, partial cds | ** | ** |
| B32P09 | FiS1A4 | BI119562 | *Pisum sativum* ssa-15 mRNA for putative senescence-associated protein, complete cds. | ** | ** |
| B32P09 | FiS2B2G10 | BM499228 | *Vigna unguiculata* phospholipase D mRNA, complete cds. | ** | ** |
| B35C12 | FiS1H23 | BI119573 | *Glycine max* ascorbate peroxidase mRNA, complete cds | ** | ** |
| B35G05 | FiS1D3 | BI245410 | *Nicotiana. tabacum* mRNA for root-specific gene. | ** | ** |
| B35L22 | FiS1G18 | BI273653 | *Glycine max* SbPRP1 gene encoding a proline-rich protein, complete cds. | ** | ** |
| B38J19 | FiS1B16 | BI119568 | EST | ** | ** |
| B38J19 | FiS1H23 | BI119573 | *Glycine max* ascorbate peroxidase mRNA, complete cds | ** | ** |
| B38J19 | FiS1i19 | BI273655 | *Glycine max* ascorbate peroxidase mRNA, complete cds. | ** | ** |
| B38J19 | FiS1M24 | BI273629 | *Solanum melongena* mRNA for QM family protein, complete cds. | ** | ** |
| B38M04 | FiS1D8 | BI118914 | *Solanum tuberosum* mRNA for plastidic ATP/ADP-transporter | ** | ** |
| B38M08 | Fi36H18 | BI347330 | *Arabidopsis thaliana* putative elongation factor 1B alpha-subunit | ** | ** |
| B38M08 | FiS1A14 | BI119552 | EST | ** | ** |
| B38M08 | FiS1B16 | BI119568 | EST | ** | ** |
| B38M08 | FiS1B4 | BI119570 | EST | ** | ** |
| B38M08 | FiS1F8 | BI273649 | EST | ** | ** |
| B38M08 | FiS1H11 | BI273656 | *Pisum sativum* ubiquitin conjugating enzyme (UBC4), complete cds. | ** | ** |
| B38M08 | FiS1J20 | BI119558 | *Cicer arietinum* partial mRNA for putative water channel protein | ** | ** |
| B38M08 | FiS1O7 | BI273688 | *Nicotiana tabacum* DNA-directed RNA polymerase IIa mRNA, complete cds. | ** | ** |
| B38M08 | FiS2B2D11 | BM499239 | *Nicotiana attenuata* pathogen-inducible alpha-dioxygenase (PIOX_NICAT) mRNA, complete cds. | ** | ** |
| B39I18 | FiS1F8 | BI273649 | EST | ** | ** |
| B42N05 | Fi36H20 | CF675620 | *Vicia faba* 5.8S, 18S and 25S ribosomal RNA genes and ITS regions | ** | ** |
| B44I13 | FiS1N22 | BI273685 | EST | ** | ** |
| B44K21 | Fi58L6 | BI347338 | EST | ** | ** |
| B45H11 | FiS1i6 | BI273669 | *Glycine max* Williams 82 lipoxygenase mRNA, complete cds. | ** | ** |
| B47H10 | Fi37A24 | BI347331 | EST | ** | ** |
| B48D20 | Fi36H20 | CF675620 | *Vicia faba* 5.8S, 18S and 25S ribosomal RNA genes and ITS regions | ** | ** |
| B48M07 | Fi36H18 | BI347330 | *Arabidopsis thaliana* putative elongation factor 1B alpha-subunit | ** | ** |
| B48M16 | FiS1J17 | BI273660 | EST | ** | ** |
| B48N15 | FiS1F1 | BI273644 | *Lupinus albus* mRNA for adenine nucleotide translocator. | ** | ** |
| B50E13 | Fi56P20 | No Acc. No. | EST | ** | ** |
| B50G06 | FiS1M6 | BI245393 | EST | ** | ** |
| B50N05 | FiS2B2E4 | BI347334 | *Glycine max* nodulin (E27) | ** | ** |
| B51J14 | FiS1H23 | BI119573 | *Glycine max* ascorbate peroxidase mRNA, complete cds | ** | ** |
| B52E15 | FiS1J24 | BI273675 | *Vigna radiata* beta galactosidase mRNA, complete cds. | ** | ** |
| B52F16 | FiS1J21 | BI273630 | *Medicago sativa* eukaryotic translation initiation factor 5A-2 mRNA, complete cds. | ** | ** |
| E05G07 | Fi36H20 | CF675620 | *Vicia faba* 5.8S, 18S and 25S ribosomal RNA genes and ITS regions | ** | ** |
| E30A07 | Fi36H20 | CF675620 | *Vicia faba* 5.8S, 18S and 25S ribosomal RNA genes and ITS regions | ** | ** |
| E48C13 | FiS1C22 | BI245396 | EST | ** | ** |
| H03L02 | FiS1i7 | BI273640 | EST | ** | ** |
| H05F07 | FiS1A14 | BI119552 | EST | ** | ** |
| H05G07 | FiS1A14 | BI119552 | EST | ** | ** |
| H06I10 | FiS1C8 | BI245400 | *Zea mays* plasma membrane integral protein ZmPIP2-7 mRNA, complete cds. | ** | ** |
| H06N12 | FiS1D5 | BI245411 | EST | ** | ** |
| H07B04 | FiS1A16 | BI119554 | *Glycine max* ADR12 mRNA | ** | ** |
| H07B04 | FiS1A17 | BI119550 | *Glycine max* actin (Soy57) gene, partial cds | ** | ** |
| H07B04 | FiS1A6 | BI119561 | *Glycine max* gene for ubiquitin, complete cds. | ** | ** |
| H07B04 | FiS1H23 | BI119573 | *Glycine max* ascorbate peroxidase mRNA, complete cds | ** | ** |
| H07B04 | FiS1i12 | BI273633 | *Oryza sativa* genomic DNA, chromosome 5, clone | ** | ** |
| H07B04 | FiS1i13 | BI273634 | EST | ** | ** |
| H07B04 | FiS1K10 | BI273670 | *Mediacgo.sativa* mRNA for peroxidase 1A. | ** | ** |
| H07B04 | FiS1K17 | BI273671 | EST | ** | ** |
| H07B04 | FiS1K18 | BI273672 | EST | ** | ** |
| H07B04 | FiS1M24 | BI273629 | *Solanum melongena* mRNA for QM family protein, complete cds. | ** | ** |
| H07B04 | FiS1N17 | BI273683 | EST | ** | ** |
| H07B04 | FiS1N21 | BI273684 | EST | ** | ** |
| H07D18 | FiS1i3 | BI273639 | EST | ** | ** |
| H08D05 | FiS1J7 | BI119563 | EST | ** | ** |
| H08D05 | FiS1N21 | BI273684 | EST | ** | ** |
| H08D19 | FiS1D1 | BI245402 | *Glycine max* peroxidase precursor (GMIPER1) mRNA, complete cds. | ** | ** |
| H08I21 | FiS1J21 | BI273630 | *Medicago sativa* eukaryotic translation initiation factor 5A-2 mRNA, complete cds. | ** | ** |
| H08I21 | FiS2B2C7 | No Acc. No. | *Glycine max* isoflavone synthase 1 (ifs1) mRNA, complete cds. | ** | ** |
| H100o03 | FiS1i18 | BI245408 | *Glycine max* mRNA for profilin, PRO1. | ** | ** |
| H100o03 | FiS2B2F4 | BM499240 | EST | ** | ** |
| H11D11 | FiS1B2 | BI119569 | EST | ** | ** |
| H12A07 | FiS1i18 | BI245408 | *Glycine max* mRNA for profilin, PRO1. | ** | ** |
| H12A07 | FiS2B2F4 | BM499240 | EST | ** | ** |
| H12A19 | FiS1A3 | BI273654 | EST | ** | ** |
| H13E15 | FiS1N3 | BI273686 | *Daucus carota* poly(A)-binding protein mRNA, complete cds. | ** | ** |
| H13N14 | FiS1B16 | BI119568 | EST | ** | ** |
| H13N14 | FiS1H23 | BI119573 | *Glycine max* ascorbate peroxidase mRNA, complete cds | ** | ** |
| H13N14 | FiS1i19 | BI273655 | *Glycine max* ascorbate peroxidase mRNA, complete cds. | ** | ** |
| H13N14 | FiS1M24 | BI273629 | *Solanum melongena* mRNA for QM family protein, complete cds. | ** | ** |
| H13N16 | FiS1B16 | BI119568 | EST | ** | ** |
| H13N16 | FiS1i19 | BI273655 | *Glycine max* ascorbate peroxidase mRNA, complete cds. | ** | ** |
| H15A05 | Fi36H18 | BI347330 | *Arabidopsis thaliana* putative elongation factor 1B alpha-subunit | ** | ** |
| H15A05 | Fi51N11 | BI347333 | Vacuolar ATP synthase | ** | ** |
| H15A05 | FiS1G17 | BI273652 | EST | ** | ** |
| H15A05 | FiS1i10 | BI119560 | *Cicer arietinum* mRNA for 20S proteasome beta subunit. | ** | ** |
| H16B17 | FiS1B10 | BI119564 | Deoxychalcone synthesis (NAD(P)H dependent 6'-deoxychalcone synthase). | ** | ** |
| H17P03 | FiS1C22 | BI245396 | EST | ** | ** |
| H17P03 | FiS1J2 | BI273662 | *Medicago sativa* mRNA for protein phosphatase 1, beta subunit. | ** | ** |
| H17P03 | FiS1J7 | BI119563 | EST | ** | ** |
| H17P03 | FiS1N21 | BI273684 | EST | ** | ** |
| H18C02 | FiS1H11 | BI273656 | *Pisum sativum* ubiquitin conjugating enzyme (UBC4), complete cds. | ** | ** |
| H21F06 | FiS1J1 | No Acc. No. | EST | ** | ** |
| H21P11 | FiS1C3 | BI245397 | *Glycine max* sucrose synthase (SS) mRNA, complete cds. | ** | ** |
| H23D11 | FiS1i6 | BI273669 | *Glycine max* Williams 82 lipoxygenase mRNA, complete cds. | ** | ** |
| H23D11 | FiS1O7 | BI273688 | *Nicotiana tabacum* DNA-directed RNA polymerase IIa mRNA, complete cds. | ** | ** |
| H23M10 | Fi58L6 | BI347338 | EST | ** | ** |
| H24N16 | FiS1B16 | BI119568 | EST | ** | ** |
| H24N16 | FiS1H23 | BI119573 | *Glycine max* ascorbate peroxidase mRNA, complete cds | ** | ** |
| H24N16 | FiS1i19 | BI273655 | *Glycine max* ascorbate peroxidase mRNA, complete cds. | ** | ** |
| H24N16 | FiS1M24 | BI273629 | *Solanum melongena* mRNA for QM family protein, complete cds. | ** | ** |
| H24P07 | FiS1C8 | BI245400 | *Zea mays* plasma membrane integral protein ZmPIP2-7 mRNA, complete cds. | ** | ** |
| H24P07 | FiS1J20 | BI119558 | *Cicer arietinum* partial mRNA for putative water channel protein | ** | ** |
| H24P07 | FiS2B2B11 | BM499231 | *Medicago truncatula* zinc transporter (ZIP) mRNA, complete cds. | ** | ** |
| H24P07 | FiS2B2C9 | BM499236 | *Glycine max* gene for ubiquitin, complete cds. | ** | ** |
| H25B11 | FiS1J23 | BI245398 | *Glycine max* farnesylated protein GMFP5 mRNA, partial cds. | ** | ** |
| H25D05 | FiS1J23 | BI245398 | *Glycine max* farnesylated protein GMFP5 mRNA, partial cds. | ** | ** |
| H25J14 | FiS1J23 | BI245398 | *Glycine max* farnesylated protein GMFP5 mRNA, partial cds. | ** | ** |
| H25K14 | FiS1i13 | BI273634 | EST | ** | ** |
| H25K14 | FiS1J23 | BI245398 | *Glycine max* farnesylated protein GMFP5 mRNA, partial cds. | ** | ** |
| H25L07 | FiS2B2F10 | CF753158 | *Sesbania rostrata* mRNA for phosphate transporter (pt1 gene). | ** | ** |
| H25L21 | FiS1J22 | BI273637 | EST | ** | ** |
| H26F07 | FiS1D24 | BI245409 | *Pisum sativum* brassinosteroid biosynthetic protein LKB (LKB) mRNA, complete cds. | ** | ** |
| H26F07 | FiS2B2D24 | BM499232 | *Glycine max* nodulin 22 gene. | ** | ** |
| H26H01 | FiS1D24 | BI245409 | *Pisum sativum* brassinosteroid biosynthetic protein LKB (LKB) mRNA, complete cds. | ** | ** |
| H26I04 | Fi51N11 | BI347333 | Vacuolar ATP synthase | ** | ** |
| H26N06 | FiS1J17 | BI273660 | EST | ** | ** |
| H26N06 | FiS1J21 | BI273630 | *Medicago sativa* eukaryotic translation initiation factor 5A-2 mRNA, complete cds. | ** | ** |
| H26N07 | FiS1J17 | BI273660 | EST | ** | ** |
| H26N07 | FiS1J21 | BI273630 | *Medicago sativa* eukaryotic translation initiation factor 5A-2 mRNA, complete cds. | ** | ** |
| H27D03 | FiS1D24 | BI245409 | *Pisum sativum* brassinosteroid biosynthetic protein LKB (LKB) mRNA, complete cds. | ** | ** |
| H27M18 | FiS1A5 | BI119557 | *Glycine max* mRNA for cinnamic acid 4-hydroxylase (CYP73). | ** | ** |
| H27O14 | FiS1i2 | BI273663 | EST | ** | ** |
| H27O14 | FiS1i8 | BI273641 | EST | ** | ** |
| H28O23 | FiS1B10 | BI119564 | Deoxychalcone synthesis (NAD(P)H dependent 6'-deoxychalcone synthase). | ** | ** |
| H29L09 | FiS1D24 | BI245409 | *Pisum sativum* brassinosteroid biosynthetic protein LKB (LKB) mRNA, complete cds. | ** | ** |
| H30C03 | FiS1G18 | BI273653 | *Glycine max* SbPRP1 gene encoding a proline-rich protein, complete cds. | ** | ** |
| H30H12 | FiS1i17 | BI273635 | EST | ** | ** |
| H33G19 | FiS2B2F10 | CF753158 | *Sesbania rostrata* mRNA for phosphate transporter (pt1 gene). | ** | ** |
| H34P05 | FiS1D24 | BI245409 | *Pisum sativum* brassinosteroid biosynthetic protein LKB (LKB) mRNA, complete cds. | ** | ** |
| H35H14 | Fi37A24 | BI347331 | EST | ** | ** |
| H36D08 | Fi36H18 | BI347330 | *Arabidopsis thaliana* putative elongation factor 1B alpha-subunit | ** | ** |
| H36D08 | FiS1G17 | BI273652 | EST | ** | ** |
| H39O23 | FiS1B2 | BI119569 | EST | ** | ** |
| H39O23 | FiS1J18 | BI273661 | *Glycine max* mRNA for nodulin-26. | ** | ** |
| H39O23 | FiS1J3 | BI273665 | *Gallus gallus* leukemia/lymohoma related factor cLRF (LRF) mRNA, complete cds. | ** | ** |
| H39O23 | FiS2B2D11 | BM499239 | *Nicotiana attenuata* pathogen-inducible alpha-dioxygenase (PIOX_NICAT) mRNA, complete cds. | ** | ** |
| H41P06 | FiS1i6 | BI273669 | *Glycine max* Williams 82 lipoxygenase mRNA, complete cds. | ** | ** |
| H41P06 | FiS1O7 | BI273688 | *Nicotiana tabacum* DNA-directed RNA polymerase IIa mRNA, complete cds. | ** | ** |
| H42H06 | FiS1D8 | BI118914 | *Solanum tuberosum* mRNA for plastidic ATP/ADP-transporter | ** | ** |
| H42K03 | Fi36H18 | BI347330 | *Arabidopsis thaliana* putative elongation factor 1B alpha-subunit | ** | ** |
| H42N09 | FiS1O7 | BI273688 | *Nicotiana tabacum* DNA-directed RNA polymerase IIa mRNA, complete cds. | ** | ** |
| H42N09 | FiS2B2D11 | BM499239 | *Nicotiana attenuata* pathogen-inducible alpha-dioxygenase (PIOX_NICAT) mRNA, complete cds. | ** | ** |
| H43E20 | FiS1B2 | BI119569 | EST | ** | ** |
| H43E20 | FiS2B2D11 | BM499239 | *Nicotiana attenuata* pathogen-inducible alpha-dioxygenase (PIOX_NICAT) mRNA, complete cds. | ** | ** |
| H43J22 | FiS1D8 | BI118914 | *Solanum tuberosum* mRNA for plastidic ATP/ADP-transporter | ** | ** |
| H43M02 | Fi55L1 | BI245395 | EST | ** | ** |
| H43M24 | Fi55C1 | BI347336 | EST | ** | ** |
| H43N13 | Fi55C1 | BI347336 | EST | ** | ** |
| H47G08 | FiS2B2F10 | CF753158 | *Sesbania rostrata* mRNA for phosphate transporter (pt1 gene). | ** | ** |
| H47J16 | Fi36H20 | CF675620 | *Vicia faba* 5.8S, 18S and 25S ribosomal RNA genes and ITS regions | ** | ** |
| H47J16 | FiS1J15 | BI273659 | EST | ** | ** |
| H49L04 | Fi58L6 | BI347338 | EST | ** | ** |
| H50N07 | FiS1K4 | BI273664 | Leghemoglobin [Psophocarpus tetragonolobus=winged-beans, nodule, mRNA, | ** | ** |
| H52B23 | FiS1H23 | BI119573 | *Glycine max* ascorbate peroxidase mRNA, complete cds | ** | ** |
| H57J19 | FiS1K17 | BI273671 | EST | ** | ** |
| H58D21 | Fi55L1 | BI245395 | EST | ** | ** |
| H59N09 | FiS1H9 | BI273631 | Pea histone H2A mRNA | ** | ** |
| H60O14 | FiS1i6 | BI273669 | *Glycine max* Williams 82 lipoxygenase mRNA, complete cds. | ** | ** |
| H61A13 | FiS1i6 | BI273669 | *Glycine max* Williams 82 lipoxygenase mRNA, complete cds. | ** | ** |
| H61L12 | FiS1i13 | BI273634 | EST | ** | ** |
| H61L12 | FiS1J16 | BI119556 | *Glycine max* nodulin 22 gene. | ** | ** |
| H64H17 | FiS1B10 | BI119564 | Deoxychalcone synthesis (NAD(P)H dependent 6'-deoxychalcone synthase). | ** | ** |
| H65D13 | FiS1D1 | BI245402 | *Glycine max* peroxidase precursor (GMIPER1) mRNA, complete cds. | ** | ** |
| H67C16 | FiS1B12 | BI119565 | *Solanum tuberosum* mRNA for putative membrane protein (poni2 gene). | ** | ** |
| H68C24 | FiS1J17 | BI273660 | EST | ** | ** |
| H68C24 | FiS2B2E4 | BM499234 | *Glycine max* nodulin (E27) | ** | ** |
| H69O23 | Fi56P20 | No Acc. No. | EST | ** | ** |
| H70I24 | FiS1i22 | BI273638 | *Daucus carota* mRNA for AX110P. | ** | ** |
| H70L21 | FiS1i13 | BI273634 | EST | ** | ** |
| H72A05 | FiS1H11 | BI273656 | *Pisum sativum* ubiquitin conjugating enzyme (UBC4), complete cds. | ** | ** |
| H73G05 | FiS1A14 | BI119552 | EST | ** | ** |
| H73G05 | FiS1i11 | BI273656 | *Stylosanthes humilis* cinnamyl alcohol dehydrogenase (CAD1) mRNA, complete cds | ** | ** |
| H73K21 | FiS1K4 | BI273664 | Leghemoglobin [Psophocarpus tetragonolobus=winged-beans, nodule, mRNA, | ** | ** |
| H74E06 | Fi55L1 | BI245395 | EST | ** | ** |
| H74E06 | FiS1i9 | BI245403 | *Phaseolus* *acutifolius* alcohol dehydrogenase-1F mRNA, complete CDS. | ** | ** |
| H76H24 | FiS1D13 | BI245414 | EST | ** | ** |
| H76L07 | FiS1H9 | BI273631 | Pea histone H2A mRNA | ** | ** |
| H80F06 | FiS1B10 | BI119564 | Deoxychalcone synthesis (NAD(P)H dependent 6'-deoxychalcone synthase). | ** | ** |
| H80P20 | FiS1J17 | BI273660 | EST | ** | ** |
| H80P20 | FiS2B2E4 | BM499234 | *Glycine max* nodulin (E27) | ** | ** |
| B51H17 | FiS1C22 | BI245396 | EST | ctg116 | Queue |
| E76J17 | FiS1C22 | BI245396 | EST | ctg116 | Queue |
| B40C23 | FiS1N22 | BI273685 | EST | ctg1261 | Queue |
| B40C23 | FiS1O1 | BI273676 | Phenylalanine ammonia-lyase [soybeans, mRNA, 1427 nt]. | ctg1261 | Queue |
| B48E15 | FiS1B16 | BI119568 | EST | ctg1277 | Queue |
| B48E15 | FiS1H23 | BI119573 | *Glycine max* ascorbate peroxidase mRNA, complete cds | ctg1277 | Queue |
| B46H23 | FiS1N17 | BI273683 | EST | ctg1350 | Queue |
| B46H23 | FiS1N21 | BI273684 | EST | ctg1350 | Queue |
| B35G12 | FiS1B14 | BI119567 | *Glycyrrhiza echinata* mRNA for O-methyltransferase, complete cds. | ctg1378 | O |
| H55O24 | FiS1G18 | BI273653 | *Glycine max* SbPRP1 gene encoding a proline-rich protein, complete cds. | ctg1474 | Queue |
| H14L10 | FiS1C3 | BI245397 | *Glycine max* sucrose synthase (SS) mRNA, complete cds. | ctg1484 | Queue |
| H12B22 | FiS1J20 | BI119558 | *Cicer arietinum* partial mRNA for putative water channel protein | ctg1519 | Queue |
| B15A19 | FiS1i7 | BI273640 | EST | ctg1632 | Queue |
| H58J12 | FiS1N17 | BI273683 | EST | ctg1633 | Queue |
| H58J12 | FiS1N21 | BI273684 | EST | ctg1633 | Queue |
| B17O12 | FiS1M22 | BI273681 | *Oryza sativa* genomic DNA, chromosome 1, PAC clone:P0408C03, complete sequence. | ctg1738 | H |
| H63N22 | FiS1A14 | BI119552 | EST | ctg1903 | Queue |
| H63N22 | FiS1i9 | BI245403 | *Phaseolus* *acutifolius* alcohol dehydrogenase-1F mRNA, complete CDS. | ctg1903 | Queue |
| H63N22 | FiS1J17 | BI273660 | EST | ctg1903 | Queue |
| H45O20 | FiS1C9 | BI245401 | *Medicago sativa* isoflavone-O-methytransferase mRNA, complete cds. | ctg191 | Queue |
| B09E12 | FiS1J16 | BI119556 | *Glycine max* nodulin 22 gene. | ctg192 | A1 |
| B09E12 | FiS1J20 | BI119558 | *Cicer arietinum* partial mRNA for putative water channel protein | ctg192 | A1 |
| H65B14 | FiS1B12 | BI119565 | *Solanum tuberosum* mRNA for putative membrane protein (poni2 gene). | ctg196 | D1AQ |
| H63N20 | FiS1A14 | BI119552 | EST | ctg202 | Queue |
| H63N20 | FiS1J17 | BI273660 | EST | ctg202 | Queue |
| H41N24 | FiS1i6 | BI273669 | *Glycine max* Williams 82 lipoxygenase mRNA, complete cds. | ctg2167 | G |
| H30G20 | FiS1i6 | BI273669 | *Glycine max* Williams 82 lipoxygenase mRNA, complete cds. | ctg218 | Queue |
| B31H04 | Fi65E19 | BI347339 | *Glycine max* myo-inositol-1-phosphate synthase (MI 1-P SYNTHASE) mRNA, complete cds. | ctg2214 | Queue |
| B31H04 | FiS1G18 | BI273653 | *Glycine max* SbPRP1 gene encoding a proline-rich protein, complete cds. | ctg2214 | Queue |
| H21M23 | Fi65E19 | BI347339 | *Glycine max* myo-inositol-1-phosphate synthase (MI 1-P SYNTHASE) mRNA, complete cds. | ctg2299 | Queue |
| H12E08 | FiS1i6 | BI273669 | *Glycine max* Williams 82 lipoxygenase mRNA, complete cds. | ctg2470 | Queue |
| B40G16 | Fi57K19 | BI347337 | EST | ctg2620 | Queue |
| H22G20 | FiS1i18 | BI245408 | *Glycine max* mRNA for profilin, PRO1. | ctg263 | Queue |
| H22G20 | FiS2B2F4 | BM499240 | EST | ctg263 | Queue |
| B45H04 | Fi55C1 | BI347336 | EST | ctg2784 | B2 & I |
| H26D04 | FiS1J20 | BI119558 | *Cicer arietinum* partial mRNA for putative water channel protein | ctg2790 | Queue |
| B54E07 | FiS1K10 | BI273670 | *Mediacgo.sativa* mRNA for peroxidase 1A. | ctg2830 | Queue |
| B54E07 | FiS1N17 | BI273683 | EST | ctg2830 | Queue |
| E57A22 | FiS1A17 | BI119550 | *Glycine max* actin (Soy57) gene, partial cds | ctg2833 | Queue |
| H26H03 | FiS1D24 | BI245409 | *Pisum sativum* brassinosteroid biosynthetic protein LKB (LKB) mRNA, complete cds. | ctg2889 | Queue |
| H26H03 | FiS1J16 | BI119556 | *Glycine max* nodulin 22 gene. | ctg2889 | Queue |
| H37L06 | FiS1O7 | BI273688 | *Nicotiana tabacum* DNA-directed RNA polymerase IIa mRNA, complete cds. | ctg3114 | Queue |
| B01H19 | FiS1B14 | BI119567 | *Glycyrrhiza echinata* mRNA for O-methyltransferase, complete cds. | ctg314 | Queue |
| H70A24 | FiS2B1D1 | No Acc. No. | *Cypripedium arietinum* mRNA for class I type 2 metallothionein (clone: CanMT-2). | ctg3192 | Queue |
| B53O21 | Fi37A24 | BI347331 | EST | ctg3196 | Queue |
| H33N21 | FiS1B16 | BI119568 | EST | ctg3198 | Queue |
| H33N21 | FiS1H23 | BI119573 | *Glycine max* ascorbate peroxidase mRNA, complete cds | ctg3198 | Queue |
| H33N21 | FiS1i19 | BI273655 | *Glycine max* ascorbate peroxidase mRNA, complete cds. | ctg3198 | Queue |
| H33N21 | FiS1M24 | BI273629 | *Solanum melongena* mRNA for QM family protein, complete cds. | ctg3198 | Queue |
| B53F09 | FiS1C8 | BI245400 | *Zea mays* plasma membrane integral protein ZmPIP2-7 mRNA, complete cds. | ctg3256 | Queue |
| B53F09 | FiS1J20 | BI119558 | *Cicer arietinum* partial mRNA for putative water channel protein | ctg3256 | Queue |
| B53F09 | FiS1K7 | BI273678 | *Arabidopsis thaliana* peroxisomal 3-keto-acyl-CoA thiolase 2 precursor (PKT2) mRNA, complete cds. | ctg3256 | Queue |
| B53F09 | FiS1M4 | BI273682 | *Glycine max* putative water channel protein (Pip1) mRNA, complete cds. | ctg3256 | Queue |
| B53F09 | FiS2B2B11 | BM499231 | *Medicago truncatula* zinc transporter (ZIP) mRNA, complete cds. | ctg3256 | Queue |
| B53F09 | FiS2B2C9 | BM499236 | *Glycine max* gene for ubiquitin, complete cds. | ctg3256 | Queue |
| H24L07 | FiS1K4 | BI273664 | Leghemoglobin [Psophocarpus tetragonolobus=winged-beans, nodule, mRNA, | ctg3310 | C2 |
| H27N04 | FiS1B10 | BI119564 | Deoxychalcone synthesis (NAD(P)H dependent 6'-deoxychalcone synthase). | ctg3464 | Queue |
| B18K03 | Fi58L6 | BI347338 | EST | ctg3475 | Queue |
| H27P14 | Fi58L6 | BI347338 | EST | ctg3475 | Queue |
| H51K24 | FiS1D5 | BI245411 | EST | ctg3515 | Queue |
| H23L03 | FiS1B16 | BI119568 | EST | ctg3558 | Queue |
| H23L03 | FiS1H23 | BI119573 | *Glycine max* ascorbate peroxidase mRNA, complete cds | ctg3558 | Queue |
| H23L03 | FiS1i19 | BI273655 | *Glycine max* ascorbate peroxidase mRNA, complete cds. | ctg3558 | Queue |
| H23L03 | FiS1M24 | BI273629 | *Solanum melongena* mRNA for QM family protein, complete cds. | ctg3558 | Queue |
| B13I24 | FiS1B16 | BI119568 | EST | ctg3619 | Queue |
| B13I24 | FiS1H23 | BI119573 | *Glycine max* ascorbate peroxidase mRNA, complete cds | ctg3619 | Queue |
| B13I24 | FiS1i19 | BI273655 | *Glycine max* ascorbate peroxidase mRNA, complete cds. | ctg3619 | Queue |
| B13I24 | FiS1M24 | BI273629 | *Solanum melongena* mRNA for QM family protein, complete cds. | ctg3619 | Queue |
| H48D17 | FiS2B2G2 | BM499242 | EST | ctg3658 | Queue |
| H20G14 | Fi36H18 | BI347330 | *Arabidopsis thaliana* putative elongation factor 1B alpha-subunit | ctg3737 | Queue |
| H16G17 | FiS1D8 | BI118914 | *Solanum tuberosum* mRNA for plastidic ATP/ADP-transporter | ctg3822 | E |
| H54N14 | FiS1K7 | BI273678 | *Arabidopsis thaliana* peroxisomal 3-keto-acyl-CoA thiolase 2 precursor (PKT2) mRNA, complete cds. | ctg3913 | Queue |
| H30M22 | FiS1i8 | BI273641 | EST | ctg3970 | Queue |
| B31G15 | Fi36H20 | CF675620 | *Vicia faba* 5.8S, 18S and 25S ribosomal RNA genes and ITS regions | ctg420 | Queue |
| H42K05 | FiS1K5 | BI273687 | *Glycine max* actin (Soy58) gene, partial cds. | ctg528 | Queue |
| B53F20 | FiS1J20 | BI119558 | *Cicer arietinum* partial mRNA for putative water channel protein | ctg599 | Queue |
| B53F20 | FiS1O7 | BI273688 | *Nicotiana tabacum* DNA-directed RNA polymerase IIa mRNA, complete cds. | ctg599 | Queue |
| H39D15 | FiS1A5 | BI119557 | *Glycine max* mRNA for cinnamic acid 4-hydroxylase (CYP73). | ctg61 | Queue |
| B48B23 | FiS1H9 | BI273631 | Pea histone H2A mRNA | ctg658 | Queue |
| H59B21 | FiS2B2F1 | CF753160 | *Vigna unguiculata* CPRD86 mRNA, partial cds. | ctg7009 | Queue |
| H77F19 | FiS1i9 | BI245403 | *Phaseolus* *acutifolius* alcohol dehydrogenase-1F mRNA, complete CDS. | ctg704 | Queue |
| B08N01 | FiS1J20 | BI119558 | *Cicer arietinum* partial mRNA for putative water channel protein | ctg8037 | Queue |
| B41D08 | Fi55C1 | BI347336 | EST | ctg8060 | Queue |
| H13K14 | FiS1J20 | BI119558 | *Cicer arietinum* partial mRNA for putative water channel protein | ctg8088 | Queue |
| H70M18 | FiS1J23 | BI245398 | *Glycine max* farnesylated protein GMFP5 mRNA, partial cds. | ctg8113 | G |
| B24B13 | FiS1C17 | BI119577 | EST | ctg8115 | Queue |
| H45A23 | FiS1B10 | BI119564 | Deoxychalcone synthesis (NAD(P)H dependent 6'-deoxychalcone synthase). | ctg8147 | Queue |
| B35O03 | FiS2B2D11 | BM499239 | *Nicotiana attenuata* pathogen-inducible alpha-dioxygenase (PIOX_NICAT) mRNA, complete cds. | ctg8158 | Queue |
| H11M05 | FiS1J23 | BI245398 | *Glycine max* farnesylated protein GMFP5 mRNA, partial cds. | ctg8185 | Queue |
| H37D14 | Fi55C1 | BI347336 | EST | ctg8207 | Queue |
| H45C14 | FiS1J17 | BI273660 | EST | ctg8223 | Queue |
| H45C14 | FiS1J21 | BI273630 | *Medicago sativa* eukaryotic translation initiation factor 5A-2 mRNA, complete cds. | ctg8223 | Queue |
| H45C14 | FiS2B2F1 | CF753160 | *Vigna unguiculata* CPRD86 mRNA, partial cds. | ctg8223 | Queue |
| E28N01 | Fi36H20 | CF675620 | *Vicia faba* 5.8S, 18S and 25S ribosomal RNA genes and ITS regions | ctg842 | Queue |
| H46N03 | FiS1G18 | BI273653 | *Glycine max* SbPRP1 gene encoding a proline-rich protein, complete cds. | ctg844 | Queue |
| E32N10 | Fi36H20 | CF675620 | *Vicia faba* 5.8S, 18S and 25S ribosomal RNA genes and ITS regions | ctg852 | Queue |
| B38J20 | FiS1B16 | BI119568 | EST | ctg9011 | G |
| H45I14 | FiS1i13 | BI273634 | EST | ctg9085 | Queue |
| H45I14 | FiS1J16 | BI119556 | *Glycine max* nodulin 22 gene. | ctg9085 | Queue |
| H45I14 | FiS1J23 | BI245398 | *Glycine max* farnesylated protein GMFP5 mRNA, partial cds. | ctg9085 | Queue |
| B35O18 | Fi37A24 | BI347331 | EST | ctg9088 | D1AQ |
| B35O18 | FiS1A16 | BI119554 | *Glycine max* ADR12 mRNA | ctg9088 | D1AQ |
| B35O18 | FiS1A17 | BI119550 | *Glycine max* actin (Soy57) gene, partial cds | ctg9088 | D1AQ |
| B35O18 | FiS1B12 | BI119565 | *Solanum tuberosum* mRNA for putative membrane protein (poni2 gene). | ctg9088 | D1AQ |
| B35O18 | FiS1H23 | BI119573 | *Glycine max* ascorbate peroxidase mRNA, complete cds | ctg9088 | D1AQ |
| B35O18 | FiS1i6 | BI273669 | *Glycine max* Williams 82 lipoxygenase mRNA, complete cds. | ctg9088 | D1AQ |
| B35O18 | FiS1J16 | BI119556 | *Glycine max* nodulin 22 gene. | ctg9088 | D1AQ |
| B31G01 | Fi65E19 | BI347339 | *Glycine max* myo-inositol-1-phosphate synthase (MI 1-P SYNTHASE) mRNA, complete cds. | ctg9102 | Queue |
| B14O12 | FiS1A3 | BI273654 | EST | ctg9105 | Queue |
| H37G08 | FiS1i18 | BI245408 | *Glycine max* mRNA for profilin, PRO1. | ctg9127 | Queue |
| H37G08 | FiS2B2F4 | BM499240 | EST | ctg9127 | Queue |
| H53H09 | FiS1O7 | BI273688 | *Nicotiana tabacum* DNA-directed RNA polymerase IIa mRNA, complete cds. | ctg9127 | Queue |
| B54F19 | Fi36H20 | CF675620 | *Vicia faba* 5.8S, 18S and 25S ribosomal RNA genes and ITS regions | ctg9140 | B1 & G |
| H07K13 | FiS1K18 | BI273672 | EST | ctg9151 | Queue |
| H44O11 | FiS1G15 | BI273650 | Alfalfa glucose-regulated endoplasmic reticular protein mRNA, complete cds. | ctg9151 | Queue |
| H78G11 | FiS1B12 | BI119565 | *Solanum tuberosum* mRNA for putative membrane protein (poni2 gene). | ctg9151 | Queue |
| H26F23 | FiS1D24 | BI245409 | *Pisum sativum* brassinosteroid biosynthetic protein LKB (LKB) mRNA, complete cds. | ctg9167 | Queue |
| B37I13 | Fi36H20 | CF675620 | *Vicia faba* 5.8S, 18S and 25S ribosomal RNA genes and ITS regions | ctg9198 | Queue |
| B37I13 | Fi65E19 | BI347339 | *Glycine max* myo-inositol-1-phosphate synthase (MI 1-P SYNTHASE) mRNA, complete cds. | ctg9198 | Queue |
| B37I13 | FiS1J22 | BI273637 | EST | ctg9198 | Queue |
| B14F03 | FiS1i6 | BI273669 | *Glycine max* Williams 82 lipoxygenase mRNA, complete cds. | ctg9202 | Queue |
| H46O24 | FiS1i6 | BI273669 | *Glycine max* Williams 82 lipoxygenase mRNA, complete cds. | ctg9202 | Queue |
| B10B20 | Fi58L6 | BI347338 | EST | ctg9210 | O |
| H42E07 | Fi56P20 | No Acc. No. | EST | ctg9223 | Queue |
| H24L08 | FiS1A12 | BI119551 | *Glycine max* chalcone synthase (chs7) gene, complete cds. | ctg9234 | Queue |
| H24L08 | FiS1J22 | BI273637 | EST | ctg9234 | Queue |
| H45E21 | FiS1i9 | BI245403 | *Phaseolus* *acutifolius* alcohol dehydrogenase-1F mRNA, complete CDS. | ctg9242 | G & M |
| H45E21 | FiS1O7 | BI273688 | *Nicotiana tabacum* DNA-directed RNA polymerase IIa mRNA, complete cds. | ctg9242 | G & M |
| H45E21 | FiS2B1D1 | No Acc. No. | *Cypripedium arietinum* mRNA for class I type 2 metallothionein (clone: CanMT-2). | ctg9242 | G & M |
| H65E02 | FiS1H23 | BI119573 | *Glycine max* ascorbate peroxidase mRNA, complete cds | ctg9256 | Queue |
| H24G04 | Fi55C1 | BI347338 | EST | ctg9272 | Queue |
| H15A06 | Fi36H18 | BI347330 | *Arabidopsis thaliana* putative elongation factor 1B alpha-subunit | ctg93 | Queue |
| H15A06 | Fi51N11 | BI347333 | Vacuolar ATP synthase | ctg93 | Queue |
| H15I03 | FiS1J15 | BI273659 | EST | ctg9301 | A1 & G |
| H15I03 | FiS1J17 | BI273660 | EST | ctg9301 | A1 & G |
| H15I03 | FiS1J21 | BI273630 | *Medicago sativa* eukaryotic translation initiation factor 5A-2 mRNA, complete cds. | ctg9301 | A1 & G |
| H15I03 | FiS1N18 | BI119551 | chalcone synthase [soybeans, mRNA, 1119 nt]. | ctg9301 | A1 & G |
| H29A08 | FiS1i6 | BI273669 | *Glycine max* Williams 82 lipoxygenase mRNA, complete cds. | ctg9306 | E |
| H31N21 | FiS1i6 | BI273669 | *Glycine max* Williams 82 lipoxygenase mRNA, complete cds. | ctg9306 | E |
| H76O12 | FiS1J20 | BI119558 | *Cicer arietinum* partial mRNA for putative water channel protein | ctg9311 | L |
| H26I22 | Fi65E19 | BI347339 | *Glycine max* myo-inositol-1-phosphate synthase (MI 1-P SYNTHASE) mRNA, complete cds. | ctg9324 | Queue |
| B23A05 | Fi36H18 | BI347330 | *Arabidopsis thaliana* putative elongation factor 1B alpha-subunit | ctg9371 | Queue |
| B23D17 | FiS1i6 | BI273669 | *Glycine max* Williams 82 lipoxygenase mRNA, complete cds. | ctg99 | Queue |

**Data not available
