# Supplementary material for: Development of a pooled probe method for locating small gene families in a physical map of soybean using stress related paralogues and a BAC minimum tile path
Source: Plant Methods. 2006 Dec 8;2:20. doi: 10.1186/1746-4811-2-20 (PMC1716159; doi:10.1186/1746-4811-2-20)
Supplement: Additional file 3 — Table of ESTs found on major linkages groups of soybean in build 4. BACs that hybridized to ESTs sorted by linkage group to identify ESTs found on the different MLGs. [file 1746-4811-2-20-S3.doc]

Additional file 3: Table of ESTs found on major linkages groups of soybean in build 4. BACs that hybridized to ESTs sorted by linkage group to identify ESTs found on the different MLGs.

| **BAC clone** | **EST (SIU ID)** | **GenBank Acc. No.** | **EST homolog** | **Contig No.** | **MLG** |
| --- | --- | --- | --- | --- | --- |
| B09E12 | Fi51P22 | BI347334 | EST | ctg192 | A1 |
| B09E12 | FiS1J16 | BI119556 | *Glycine max* nodulin 22 gene. | ctg192 | A1 |
| B09E12 | FiS1J20 | BI119558 | *Cicer arietinum* partial mRNA for putative water channel protein | ctg192 | A1 |
| H15I03 | FiS1J15 | BI273659 | EST | ctg9301 | A1 & G |
| H15I03 | FiS1J17 | BI273660 | EST | ctg9301 | A1 & G |
| H15I03 | FiS1J21 | BI273630 | *Medicago sativa* eukaryotic translation initiation factor 5A-2 mRNA, complete cds. | ctg9301 | A1 & G |
| H15I03 | FiS1N18 | BI119551 | chalcone synthase [soybeans, mRNA, 1119 nt]. | ctg9301 | A1 & G |
| B24C11 | Gm-r1021-3B19 | AI443896 | Aspartate aminotransferase 1 | ctg2704 | A2 & C2 |
| B36N10 | Gm-r1021-6M24 | AI461071 | Beta tubulin | ctg9161 | B1 |
| B54F19 | Fi36H20 | CF675620 | *Vicia faba* 5.8S, 18S and 25S ribosomal RNA genes and ITS regions | ctg9140 | B1 & G |
| B45H04 | Fi55C1 | BI347336 | EST | ctg2784 | B2 & I |
| H22H05 | Gm-r1021-3H12 | AI441809 | Beta-galactosidase | ctg3036 | C2 |
| H22H05 | Gm-r1021-6D10 | AI460671 | Kinesinlike protein A | ctg3036 | C2 |
| H24L07 | FiS1K4 | BI273664 | Leghemoglobin [Psophocarpus tetragonolobus=winged-beans, nodule, mRNA, | ctg3310 | C2 |
| H57O09 | Gm-r1021-5K20 | AI440721 | MAP kinase kinase alpha protein kinase | ctg9357 | C2 |
| H57O09 | Gm-R1021-6J19 | AI460618 | Calmodulin-stimulated calcium ATPase | ctg9357 | C2 |
| H65B14 | FiS1B12 | BI119565 | *Solanum tuberosum* mRNA for putative membrane protein (poni2 gene). | ctg196 | D1AQ |
| H46H10 | Gm-r1021-2L12 | AI437773 | Cytochrome P450 | ctg8188 | D1AQ |
| B35O18 | Fi37A24 | BI347331 | EST | ctg9088 | D1AQ |
| B35O18 | FiS1A16 | BI119554 | *Glycine max* ADR12 mRNA | ctg9088 | D1AQ |
| B35O18 | FiS1A17 | BI119550 | *Glycine max* actin (Soy57) gene, partial cds | ctg9088 | D1AQ |
| B35O18 | FiS1B12 | BI119565 | *Solanum tuberosum* mRNA for putative membrane protein (poni2 gene). | ctg9088 | D1AQ |
| B35O18 | FiS1H23 | BI119573 | *Glycine max* ascorbate peroxidase mRNA, complete cds | ctg9088 | D1AQ |
| B35O18 | FiS1i6 | BI273669 | *Glycine max* Williams 82 lipoxygenase mRNA, complete cds. | ctg9088 | D1AQ |
| B35O18 | FiS1J16 | BI119556 | *Glycine max* nodulin 22 gene. | ctg9088 | D1AQ |
| H77N20 | Gm-r1021-2C08 | AI437977 | Protein disulfide isomerase | ctg9292 | D1AQ |
| H44P15 | Gm-r1021-2I23 | AI437632 | 4-Coumarate CoA ligase 1 | ctg9361 | D1BW |
| H16G17 | FiS1D8 | BI118914 | *Solanum tuberosum* mRNA for plastidic ATP/ADP-transporter | ctg3822 | E |
| H29A08 | FiS1i6 | BI273669 | *Glycine max* Williams 82 lipoxygenase mRNA, complete cds. | ctg9306 | E |
| H31N21 | FiS1i6 | BI273669 | *Glycine max* Williams 82 lipoxygenase mRNA, complete cds. | ctg9306 | E |
| H33O15 | Gm-r1021-2H06 | AI437704 | Cellulose synthase | ctg9349 | E |
| H41N24 | FiS1i6 | BI273669 | *Glycine max* Williams 82 lipoxygenase mRNA, complete cds. | ctg2167 | G |
| H70M18 | FiS1J23 | BI245398 | *Glycine max* farnesylated protein GMFP5 mRNA, partial cds. | ctg8113 | G |
| B38J20 | FiS1B16 | BI119568 | EST | ctg9011 | G |
| H77P02 | Gm-r1021-7D20 | AI441758 | Flavonol synthase | ctg9354 | G |
| H45E21 | FiS1i9 | BI245403 | *Phaseolus* *acutifolius* alcohol dehydrogenase-1F mRNA, complete CDS. | ctg9242 | G & M |
| H45E21 | FiS1O7 | BI273688 | *Nicotiana tabacum* DNA-directed RNA polymerase IIa mRNA, complete cds. | ctg9242 | G & M |
| H45E21 | FiS2B1D1 | No Acc. No. | *Cypripedium arietinum* mRNA for class I type 2 metallothionein (clone: CanMT-2). | ctg9242 | G & M |
| H35I01 | Gm-r1021-3K03 | AI442658 | Calcium binding protein isolog | ctg1120 | H |
| H35I01 | Gm-r1021-5J01 | AI441021 | Calciumdependent protein kinase | ctg1120 | H |
| B17O12 | FiS1M22 | BI273681 | *Oryza sativa* genomic DNA, chromosome 1, PAC clone:P0408C03, complete sequence. | ctg1738 | H |
| H06B23 | Gm-r1021-2F03 | AI437497 | Calretulin | ctg9056 | I |
| H24J01 | Gm-r1021-1P11 | AI444099 | 3-Isopropylmalate dehydratase, leud subunit. | ctg641 | J |
| H19B13 | Gm-r1021-4D05 | AI440931 | 1-Aminocyclopropane-1-carboxylate oxidase | ctg2108 | K |
| H76O12 | FiS1J20 | BI119558 | *Cicer arietinum* partial mRNA for putative water channel protein | ctg9311 | L |
| B35G12 | FiS1B14 | BI119567 | *Glycyrrhiza echinata* mRNA for O-methyltransferase, complete cds. | ctg1378 | O |
| B10B20 | Fi58L6 | BI347338 | EST | ctg9210 | O |
| B51L05 | Gm-r1021-5J01 | AI441021 | Calciumdependent protein kinase | ctg9210 | O |
| B02G20 | Gm-r1021-6J24 | AI522819 | Waterstress induced tonoplast intrinsic protein | ctg1079 | Queue |
| B51H17 | FiS1C22 | BI245396 | EST | ctg116 | Queue |
| E76J17 | FiS1C22 | BI245396 | EST | ctg116 | Queue |
| B40C23 | FiS1N22 | BI273685 | EST | ctg1261 | Queue |
| B40C23 | FiS1O1 | BI273676 | Phenylalanine ammonia-lyase [soybeans, mRNA, 1427 nt]. | ctg1261 | Queue |
| B48E15 | FiS1B16 | BI119568 | EST | ctg1277 | Queue |
| B48E15 | FiS1H23 | BI119573 | *Glycine max* ascorbate peroxidase mRNA, complete cds | ctg1277 | Queue |
| B46H23 | FiS1N17 | BI273683 | EST | ctg1350 | Queue |
| B46H23 | FiS1N21 | BI273684 | EST | ctg1350 | Queue |
| H31J02 | Gm-r1021-2B16 | AI437902 | Threonine synthase | ctg1443 | Queue |
| H31J02 | Gm-r1021-2E06 | AI437535 | Quinone oxidoreductase | ctg1443 | Queue |
| H31J02 | Gm-r1021-3H12 | AI441809 | Beta-galactosidase | ctg1443 | Queue |
| H31J02 | Gm-r1021-5K20 | AI440721 | MAP kinase kinase alpha protein kinase | ctg1443 | Queue |
| H31J02 | Gm-r1021-6D10 | AI460671 | Kinesinlike protein A | ctg1443 | Queue |
| H55O24 | FiS1G18 | BI273653 | *Glycine max* SbPRP1 gene encoding a proline-rich protein, complete cds. | ctg1474 | Queue |
| H14L10 | FiS1C3 | BI245397 | *Glycine max* sucrose synthase (SS) mRNA, complete cds. | ctg1484 | Queue |
| H12B22 | FiS1J20 | BI119558 | *Cicer arietinum* partial mRNA for putative water channel protein | ctg1519 | Queue |
| B15A19 | FiS1i7 | BI273640 | EST | ctg1632 | Queue |
| H58J12 | FiS1N17 | BI273683 | EST | ctg1633 | Queue |
| H58J12 | FiS1N21 | BI273684 | EST | ctg1633 | Queue |
| H39A16 | Gm-r1021-1D08 | AI443444 | Proline-rich 14 KDA protein | ctg1751 | Queue |
| H39A16 | Gm-r1021-1M21 | AI442373 | 4-Coumarate:CoA ligase isoform 2 | ctg1751 | Queue |
| H39A16 | Gm-r1021-2B16 | AI437902 | Threonine synthase | ctg1751 | Queue |
| H39A16 | Gm-r1021-2C03 | AI444115 | 7-O-methyltransferase | ctg1751 | Queue |
| H39A16 | Gm-r1021-2C08 | AI437977 | Protein disulfide isomerase | ctg1751 | Queue |
| H39A16 | Gm-r1021-2C14 | AI437618 | Glycine cleavage system H protein precursor | ctg1751 | Queue |
| H39A16 | Gm-r1021-2E06 | AI437535 | Quinone oxidoreductase | ctg1751 | Queue |
| H39A16 | Gm-r1021-2F06 | AI437703 | Calmodulin | ctg1751 | Queue |
| H39A16 | Gm-r1021-2F09 | AI438014 | Epoxide hydrolase | ctg1751 | Queue |
| H39A16 | Gm-r1021-3H12 | AI441809 | Beta-galactosidase | ctg1751 | Queue |
| H39A16 | Gm-r1021-5J22 | AI442296 | Calmodulinlike protein | ctg1751 | Queue |
| H39A16 | Gm-r1021-5K20 | AI440721 | MAP kinase kinase alpha protein kinase | ctg1751 | Queue |
| H39A16 | Gm-r1021-6D13 | AI461073 | CLV1 receptor kinase | ctg1751 | Queue |
| H39A16 | Gm-R1021-6J19 | AI460618 | Calmodulin-stimulated calcium ATPase | ctg1751 | Queue |
| H39A16 | Gm-r1021-7D02 | AI442731 | Casein kinase II beta chain | ctg1751 | Queue |
| H63N22 | FiS1A14 | BI119552 | EST | ctg1903 | Queue |
| H63N22 | FiS1i9 | BI245403 | *Phaseolus* *acutifolius* alcohol dehydrogenase-1F mRNA, complete CDS. | ctg1903 | Queue |
| H63N22 | FiS1J17 | BI273660 | EST | ctg1903 | Queue |
| H45O20 | Gm-r1021-2C03 | AI444115 | 7-O-methyltransferase | ctg191 | Queue |
| H45O20 | FiS1C9 | BI245401 | *Medicago sativa* isoflavone-O-methytransferase mRNA, complete cds. | ctg191 | Queue |
| H63N20 | FiS1A14 | BI119552 | EST | ctg202 | Queue |
| H63N20 | FiS1J17 | BI273660 | EST | ctg202 | Queue |
| H30G20 | FiS1i6 | BI273669 | *Glycine max* Williams 82 lipoxygenase mRNA, complete cds. | ctg218 | Queue |
| B31H04 | Fi65E19 | BI347339 | *Glycine max* myo-inositol-1-phosphate synthase (MI 1-P SYNTHASE) mRNA, complete cds. | ctg2214 | Queue |
| B31H04 | FiS1G18 | BI273653 | *Glycine max* SbPRP1 gene encoding a proline-rich protein, complete cds. | ctg2214 | Queue |
| H21M23 | Fi65E19 | BI347339 | *Glycine max* myo-inositol-1-phosphate synthase (MI 1-P SYNTHASE) mRNA, complete cds. | ctg2299 | Queue |
| H12E08 | FiS1i6 | BI273669 | *Glycine max* Williams 82 lipoxygenase mRNA, complete cds. | ctg2470 | Queue |
| B40G16 | Fi57K19 | BI347337 | EST | ctg2620 | Queue |
| H22G20 | FiS1i18 | BI245408 | *Glycine max* mRNA for profilin, PRO1. | ctg263 | Queue |
| H22G20 | FiS2B2F4 | BM499240 | EST | ctg263 | Queue |
| B23M01 | Gm-r1021-3H22 | AI443248 | Calnexin | ctg2657 | Queue |
| B23M01 | Gm-r1021-4P01 | AI440894 | Serine/threonine kinase | ctg2657 | Queue |
| B23M01 | Gm-r1021-5B03 | AI440630 | Putative potassium transporter ATKT2P | ctg2657 | Queue |
| B23M01 | Gm-r1021-6H24 | AI461080 | Ethylene-forming enzyme | ctg2657 | Queue |
| H26D04 | FiS1J20 | BI119558 | *Cicer arietinum* partial mRNA for putative water channel protein | ctg2790 | Queue |
| B54E07 | FiS1K10 | BI273670 | *Mediacgo.sativa* mRNA for peroxidase 1A. | ctg2830 | Queue |
| B54E07 | FiS1N17 | BI273683 | EST | ctg2830 | Queue |
| E57A22 | FiS1A17 | BI119550 | *Glycine max* actin (Soy57) gene, partial cds | ctg2833 | Queue |
| H26H03 | FiS1D24 | BI245409 | *Pisum sativum* brassinosteroid biosynthetic protein LKB (LKB) mRNA, complete cds. | ctg2889 | Queue |
| H26H03 | FiS1J16 | BI119556 | *Glycine max* nodulin 22 gene. | ctg2889 | Queue |
| H18A04 | Gm-r1021-2E16 | AI437503 | Indole-3-acetate beta-glucosyltransferase isolog | ctg2892 | Queue |
| H63O23 | Gm-r1021-6D10 | AI460671 | Kinesinlike protein A | ctg2988 | Queue |
| H37L06 | FiS1O7 | BI273688 | *Nicotiana tabacum* DNA-directed RNA polymerase IIa mRNA, complete cds. | ctg3114 | Queue |
| B01H19 | FiS1B14 | BI119567 | *Glycyrrhiza echinata* mRNA for O-methyltransferase, complete cds. | ctg314 | Queue |
| H70A24 | FiS2B1D1 | No Acc. No. | *Cypripedium arietinum* mRNA for class I type 2 metallothionein (clone: CanMT-2). | ctg3192 | Queue |
| B53O21 | Fi37A24 | BI347331 | EST | ctg3196 | Queue |
| H33N21 | FiS1B16 | BI119568 | EST | ctg3198 | Queue |
| H33N21 | FiS1H23 | BI119573 | *Glycine max* ascorbate peroxidase mRNA, complete cds | ctg3198 | Queue |
| H33N21 | FiS1i19 | BI273655 | *Glycine max* ascorbate peroxidase mRNA, complete cds. | ctg3198 | Queue |
| H33N21 | FiS1M24 | BI273629 | *Solanum melongena* mRNA for QM family protein, complete cds. | ctg3198 | Queue |
| B53F09 | Gm-r1021-2C08 | AI437977 | Protein disulfide isomerase | ctg3256 | Queue |
| B53F09 | FiS1C8 | BI245400 | *Zea mays* plasma membrane integral protein ZmPIP2-7 mRNA, complete cds. | ctg3256 | Queue |
| B53F09 | FiS1J20 | BI119558 | *Cicer arietinum* partial mRNA for putative water channel protein | ctg3256 | Queue |
| B53F09 | FiS1K7 | BI273678 | *Arabidopsis thaliana* peroxisomal 3-keto-acyl-CoA thiolase 2 precursor (PKT2) mRNA, complete cds. | ctg3256 | Queue |
| B53F09 | FiS1M4 | BI273682 | *Glycine max* putative water channel protein (Pip1) mRNA, complete cds. | ctg3256 | Queue |
| B53F09 | FiS2B2B11 | BM499231 | *Medicago truncatula* zinc transporter (ZIP) mRNA, complete cds. | ctg3256 | Queue |
| B53F09 | FiS2B2C9 | BM499236 | *Glycine max* gene for ubiquitin, complete cds. | ctg3256 | Queue |
| B40B12 | Gm-r1021-6D10 | AI460671 | Kinesinlike protein A | ctg3303 | Queue |
| H42M11 | Gm-r1021-2H06 | AI437704 | Cellulose synthase | ctg3333 | Queue |
| H27N04 | FiS1B10 | BI119564 | Deoxychalcone synthesis (NAD(P)H dependent 6'-deoxychalcone synthase). | ctg3464 | Queue |
| B18K03 | Fi58L6 | BI347338 | EST | ctg3475 | Queue |
| H27P14 | Fi58L6 | BI347338 | EST | ctg3475 | Queue |
| H51K24 | FiS1D5 | BI245411 | EST | ctg3515 | Queue |
| B38E05 | Gm-r1021-5I21 | AI441043 | Vacuolar ATP synthase catalytic subunit A | ctg3520 | Queue |
| H23L03 | FiS1B16 | BI119568 | EST | ctg3558 | Queue |
| H23L03 | FiS1H23 | BI119573 | *Glycine max* ascorbate peroxidase mRNA, complete cds | ctg3558 | Queue |
| H23L03 | FiS1i19 | BI273655 | *Glycine max* ascorbate peroxidase mRNA, complete cds. | ctg3558 | Queue |
| H23L03 | FiS1M24 | BI273629 | *Solanum melongena* mRNA for QM family protein, complete cds. | ctg3558 | Queue |
| B13I24 | FiS1B16 | BI119568 | EST | ctg3619 | Queue |
| B13I24 | FiS1H23 | BI119573 | *Glycine max* ascorbate peroxidase mRNA, complete cds | ctg3619 | Queue |
| B13I24 | FiS1i19 | BI273655 | *Glycine max* ascorbate peroxidase mRNA, complete cds. | ctg3619 | Queue |
| B13I24 | FiS1M24 | BI273629 | *Solanum melongena* mRNA for QM family protein, complete cds. | ctg3619 | Queue |
| H48D17 | FiS2B2G2 | BM499242 | EST | ctg3658 | Queue |
| B50A20 | Gm-r1021-2E16 | AI437503 | Indole-3-acetate beta-glucosyltransferase isolog | ctg3687 | Queue |
| H20G14 | Fi36H18 | BI347330 | *Arabidopsis thaliana* putative elongation factor 1B alpha-subunit | ctg3737 | Queue |
| H54N14 | FiS1K7 | BI273678 | *Arabidopsis thaliana* peroxisomal 3-keto-acyl-CoA thiolase 2 precursor (PKT2) mRNA, complete cds. | ctg3913 | Queue |
| H30M22 | FiS1i8 | BI273641 | EST | ctg3970 | Queue |
| B31G15 | Fi36H20 | CF675620 | *Vicia faba* 5.8S, 18S and 25S ribosomal RNA genes and ITS regions | ctg420 | Queue |
| H42K05 | FiS1K5 | BI273687 | *Glycine max* actin (Soy58) gene, partial cds. | ctg528 | Queue |
| H20A11 | Gm-r1021-4B19 | AI440861 | 3-Deoxy-D-arabino-heptulosonate 7-phosphate synthase | ctg542 | Queue |
| H76D11 | Gm-R1021-4C18 | AI443166 | Pectate lyase | ctg558 | Queue |
| H76D11 | Gm-r1021-5H08 | AI441087 | Protein kinase | ctg558 | Queue |
| B53F20 | FiS1J20 | BI119558 | *Cicer arietinum* partial mRNA for putative water channel protein | ctg599 | Queue |
| B53F20 | FiS1O7 | BI273688 | *Nicotiana tabacum* DNA-directed RNA polymerase IIa mRNA, complete cds. | ctg599 | Queue |
| H39D15 | Gm-r1021-3H12 | AI441809 | Beta-galactosidase | ctg61 | Queue |
| H39D15 | FiS1A5 | BI119557 | *Glycine max* mRNA for cinnamic acid 4-hydroxylase (CYP73). | ctg61 | Queue |
| H41O12 | Gm-r1021-10H24 | AI495635 | Casein kinase II, alpha chain | ctg646 | Queue |
| B48B23 | FiS1H9 | BI273631 | Pea histone H2A mRNA | ctg658 | Queue |
| H59B21 | FiS2B2F1 | CF753160 | *Vigna unguiculata* CPRD86 mRNA, partial cds. | ctg7009 | Queue |
| H77F19 | FiS1i9 | BI245403 | *Phaseolus* *acutifolius* alcohol dehydrogenase-1F mRNA, complete CDS. | ctg704 | Queue |
| B08N01 | FiS1J20 | BI119558 | *Cicer arietinum* partial mRNA for putative water channel protein | ctg8037 | Queue |
| B41D08 | Fi55C1 | BI347336 | EST | ctg8060 | Queue |
| H13K14 | FiS1J20 | BI119558 | *Cicer arietinum* partial mRNA for putative water channel protein | ctg8088 | Queue |
| B24B13 | FiS1C17 | BI119577 | EST | ctg8115 | Queue |
| H66A06 | Gm-r1021-5J22 | AI442296 | Calmodulinlike protein | ctg8120 | Queue |
| H66A06 | Gm-R1021-6J19 | AI460618 | Calmodulin-stimulated calcium ATPase | ctg8120 | Queue |
| H45A23 | FiS1B10 | BI119564 | Deoxychalcone synthesis (NAD(P)H dependent 6'-deoxychalcone synthase). | ctg8147 | Queue |
| B35O03 | FiS2B2D11 | BM499239 | *Nicotiana attenuata* pathogen-inducible alpha-dioxygenase (PIOX_NICAT) mRNA, complete cds. | ctg8158 | Queue |
| H11M05 | FiS1J23 | BI245398 | *Glycine max* farnesylated protein GMFP5 mRNA, partial cds. | ctg8185 | Queue |
| H37D14 | Fi55C1 | BI347336 | EST | ctg8207 | Queue |
| H45C14 | FiS1J17 | BI273660 | EST | ctg8223 | Queue |
| H45C14 | FiS1J21 | BI273630 | *Medicago sativa* eukaryotic translation initiation factor 5A-2 mRNA, complete cds. | ctg8223 | Queue |
| H45C14 | FiS2B2F1 | CF753160 | *Vigna unguiculata* CPRD86 mRNA, partial cds. | ctg8223 | Queue |
| E28N01 | Fi36H20 | CF675620 | *Vicia faba* 5.8S, 18S and 25S ribosomal RNA genes and ITS regions | ctg842 | Queue |
| H46N03 | FiS1G18 | BI273653 | *Glycine max* SbPRP1 gene encoding a proline-rich protein, complete cds. | ctg844 | Queue |
| E32N10 | Fi36H20 | CF675620 | *Vicia faba* 5.8S, 18S and 25S ribosomal RNA genes and ITS regions | ctg852 | Queue |
| B46F09 | Gm-r1021-6D10 | AI460671 | Kinesinlike protein A | ctg858 | Queue |
| H45M23 | Gm-r1021-6D10 | AI460671 | Kinesinlike protein A | ctg858 | Queue |
| H45M23 | Gm-r1021-6J24 | AI522819 | Waterstress induced tonoplast intrinsic protein | ctg858 | Queue |
| H13E14 | Gm-r1021-3N22 | AI442245 | Vacuolar ATP synthase subunit C | ctg9034 | Queue |
| H39D19 | Gm-r1021-3K03 | AI442658 | Calcium binding protein isolog | ctg9048 | Queue |
| H45I14 | FiS1i13 | BI273634 | EST | ctg9085 | Queue |
| H45I14 | FiS1J16 | BI119556 | *Glycine max* nodulin 22 gene. | ctg9085 | Queue |
| H45I14 | FiS1J23 | BI245398 | *Glycine max* farnesylated protein GMFP5 mRNA, partial cds. | ctg9085 | Queue |
| B31G01 | Fi65E19 | BI347339 | *Glycine max* myo-inositol-1-phosphate synthase (MI 1-P SYNTHASE) mRNA, complete cds. | ctg9102 | Queue |
| B14O12 | FiS1A3 | BI273654 | EST | ctg9105 | Queue |
| H37G08 | FiS1i18 | BI245408 | *Glycine max* mRNA for profilin, PRO1. | ctg9127 | Queue |
| H37G08 | FiS2B2F4 | BM499240 | EST | ctg9127 | Queue |
| H53H09 | FiS1O7 | BI273688 | *Nicotiana tabacum* DNA-directed RNA polymerase IIa mRNA, complete cds. | ctg9127 | Queue |
| H07K13 | FiS1K18 | BI273672 | EST | ctg9151 | Queue |
| H44O11 | FiS1G15 | BI273650 | Alfalfa glucose-regulated endoplasmic reticular protein mRNA, complete cds. | ctg9151 | Queue |
| H78G11 | FiS1B12 | BI119565 | *Solanum tuberosum* mRNA for putative membrane protein (poni2 gene). | ctg9151 | Queue |
| H26F23 | FiS1D24 | BI245409 | *Pisum sativum* brassinosteroid biosynthetic protein LKB (LKB) mRNA, complete cds. | ctg9167 | Queue |
| B37I13 | Fi36H20 | CF675620 | *Vicia faba* 5.8S, 18S and 25S ribosomal RNA genes and ITS regions | ctg9198 | Queue |
| B37I13 | Fi65E19 | BI347339 | *Glycine max* myo-inositol-1-phosphate synthase (MI 1-P SYNTHASE) mRNA, complete cds. | ctg9198 | Queue |
| B37I13 | FiS1J22 | BI273637 | EST | ctg9198 | Queue |
| B31L10 | Gm-r1021-3I07 | AI442627 | Aminotransferase | ctg9201 | Queue |
| B14F03 | FiS1i6 | BI273669 | *Glycine max* Williams 82 lipoxygenase mRNA, complete cds. | ctg9202 | Queue |
| H46O24 | FiS1i6 | BI273669 | *Glycine max* Williams 82 lipoxygenase mRNA, complete cds. | ctg9202 | Queue |
| H42E07 | Fi56P20 | No Acc. No. | EST | ctg9223 | Queue |
| H24L08 | FiS1A12 | BI119551 | *Glycine max* chalcone synthase (chs7) gene, complete cds. | ctg9234 | Queue |
| H24L08 | FiS1J22 | BI273637 | EST | ctg9234 | Queue |
| H58E12 | Gm-r1021-2E06 | AI437535 | Quinone oxidoreductase | ctg9240 | Queue |
| H58E12 | Gm-r1021-3H12 | AI441809 | Beta-galactosidase | ctg9240 | Queue |
| H65E02 | FiS1H23 | BI119573. | *Glycine max* ascorbate peroxidase mRNA, complete cds | ctg9256 | Queue |
| H40B02 | Gm-r1021-1B10 | AI444067 | Ca2+ ATPase | ctg9271 | Queue |
| H24G04 | Fi55C1 | BI347338 | EST | ctg9272 | Queue |
| H15A06 | Fi36H18 | BI347330 | *Arabidopsis thaliana* putative elongation factor 1B alpha-subunit | ctg93 | Queue |
| H15A06 | Fi51N11 | BI347333 | Vacuolar ATP synthase | ctg93 | Queue |
| H35G05 | Gm-r1021-5J01 | AI441021 | Calciumdependent protein kinase | ctg9310 | Queue |
| B14M21 | Gm-r1021-2F06 | AI437703 | Calmodulin | ctg9320 | Queue |
| B14M21 | Gm-r1021-7D02 | AI442731 | Casein kinase II beta chain | ctg9320 | Queue |
| B14M21 | Gm-r1021-7D20 | AI441758 | Flavonol synthase | ctg9320 | Queue |
| H26I22 | Fi65E19 | BI347339 | *Glycine max* myo-inositol-1-phosphate synthase (MI 1-P SYNTHASE) mRNA, complete cds. | ctg9324 | Queue |
| H41O05 | Gm-r1021-3H12 | AI441809 | Beta-galactosidase | ctg9340 | Queue |
| B23A05 | Fi36H18 | BI347330 | *Arabidopsis thaliana* putative elongation factor 1B alpha-subunit | ctg9371 | Queue |
| B07L16 | Gm-r1021-5J22 | AI442296 | Calmodulinlike protein | ctg9376 | Queue |
| B07L16 | Gm-r1021-6J19 | AI460618 | Calmodulin-stimulated calcium ATPase | ctg9376 | Queue |
| B23D17 | FiS1i6 | BI273669 | *Glycine max* Williams 82 lipoxygenase mRNA, complete cds. | ctg99 | Queue |
