# Supplementary material for: Development of a pooled probe method for locating small gene families in a physical map of soybean using stress related paralogues and a BAC minimum tile path
Source: Plant Methods. 2006 Dec 8;2:20. doi: 10.1186/1746-4811-2-20 (PMC1716159; doi:10.1186/1746-4811-2-20)
Supplement: Additional file 4 — Locations inferred in the build 3 version of the physical map of BAC clones hybridized to subtracted ESTs showing contig number and linkage group if determined. BACs that hybridized to ESTs in build 3 sorted by linkage group to identify ESTs found on the different MLGs. [file 1746-4811-2-20-S4.doc]

Additional file 4: Locations inferred in the build 3 version of the physical map of BAC clones hybridized to subtracted ESTs showing contig number and linkage group if determined. BACs that hybridized to ESTs in build 3 sorted by linkage group to identify ESTs found on the different MLGs.

| **BAC CLONE** | **EST** | **GenBank gi #** | **Annotated as sharing homology with** | **Contig and location** |
| --- | --- | --- | --- | --- |
| B23C13 | Fi36H18 | BI347330 | *Arabidopsis thaliana* putative elongation factor 1B alpha-subunit | ctg515, D1AQ |
| B48M07 | Fi36H18 | BI347330 | *Arabidopsis thaliana* putative elongation factor 1B alpha-subunit | ctg6, H |
| H20G14 | Fi36H18 | BI347330 | *Arabidopsis thaliana* putative elongation factor 1B alpha-subunit | Queue, ctg1541 |
| H15A05 | Fi36H18 | BI347330 | *Arabidopsis thaliana* putative elongation factor 1B alpha-subunit | Queue, ctg1961 |
| B38M08 | Fi36H18 | BI347330 | *Arabidopsis thaliana* putative elongation factor 1B alpha-subunit | Queue, ctg206 |
| H36D08 | Fi36H18 | BI347330 | *Arabidopsis thaliana* putative elongation factor 1B alpha-subunit | Queue, ctg245 |
| B23A05 | Fi36H18 | BI347330 | *Arabidopsis thaliana* putative elongation factor 1B alpha-subunit | Queue, ctg574 |
| H42K03 | Fi36H18 | BI347330 | *Arabidopsis thaliana* putative elongation factor 1B alpha-subunit | Queue, ctg682 |
| H15A06 | Fi36H18 | BI347330 | *Arabidopsis thaliana* putative elongation factor 1B alpha-subunit | Sd wt 6-4, ctg479, A1 |
| B37I13 | Fi36H20 | No Acc. # | *Vicia faba* 5.8S, 18S and 25S ribosomal RNA genes and ITS regions | ctg115, Pod mat 5-4, K |
| B48D20 | Fi36H20 | No Acc. # | *Vicia faba* 5.8S, 18S and 25S ribosomal RNA genes and ITS regions | ctg539, A2 |
| H47J16 | Fi36H20 | No Acc. # | *Vicia faba* 5.8S, 18S and 25S ribosomal RNA genes and ITS regions | ctg913, Queue |
| B42N05 | Fi36H20 | No Acc. # | *Vicia faba* 5.8S, 18S and 25S ribosomal RNA genes and ITS regions | Queue, ctg104 |
| B54F19 | Fi36H20 | No Acc. # | *Vicia faba* 5.8S, 18S and 25S ribosomal RNA genes and ITS regions | Queue, ctg389 |
| B31G15 | Fi36H20 | No Acc. # | *Vicia faba* 5.8S, 18S and 25S ribosomal RNA genes and ITS regions | Queue, ctg51 |
| E05G07 | Fi36H20 | No Acc. # | *Vicia faba* 5.8S, 18S and 25S ribosomal RNA genes and ITS regions |  |
| E30A07 | Fi36H20 | No Acc. # | *Vicia faba* 5.8S, 18S and 25S ribosomal RNA genes and ITS regions |  |
| E32N10 | Fi36H20 | No Acc. # | *Vicia faba* 5.8S, 18S and 25S ribosomal RNA genes and ITS regions |  |
| E28N01 | Fi36H20 | No Acc. # | *Vicia faba* 5.8S, 18S and 25S ribosomal RNA genes and ITS regions |  |
| B03M12 | Fi37A24 | BI347331 | EST | Queue, ctg1621 |
| B04A07 | Fi37A24 | BI347331 | EST | Queue, ctg1668 |
| B35O18 | Fi37A24 | BI347331 | EST | Queue, ctg287 |
| B53O21 | Fi37A24 | BI347331 | EST | Queue, ctg519 |
| B32C13 | Fi37A24 | BI347331 | EST | Queue, ctg736 |
| B47H10 | Fi37A24 | BI347331 | EST | Queue, ctg790 |
| H35H14 | Fi37A24 | BI347331 | EST | Queue, ctg790 |
| B14I07 | Fi39D23 | BI347332 | *Glycine max* ADR12 mRNA | Queue, ctg1883 |
| H15A05 | Fi51N11 | BI347333 | Vacuolar ATP synthase | Queue, ctg1961 |
| H26I04 | Fi51N11 | BI347333 | Vacuolar ATP synthase | Queue, ctg682 |
| H15A06 | Fi51N11 | BI347333 | Vacuolar ATP synthase | Sd wt 6-4, ctg479, A1 |
| H43M24 | Fi55C1 |  | EST | ctg189, D1AQ |
| H24G04 | Fi55C1 |  | EST | ctg933, Queue |
| B41D08 | Fi55C1 |  | EST | Queue, ctg173 |
| H37D14 | Fi55C1 |  | EST | Queue, ctg627 |
| B45H04 | Fi55C1 |  | EST | Queue, pB039c, pA043a, pA109d, ctg742 |
| H43N13 | Fi55C1 |  | EST |  |
| H74E06 | Fi55L1 | BI245395 | EST | ctg924, Queue |
| H58D21 | Fi55L1 | BI245395 | EST | Queue, ctg35 |
| H43M02 | Fi55L1 | BI245395 | EST | Queue, pA481a, ctg1863 |
| B50E13 | Fi56P20 |  | EST | Queue, ctg1243 |
| H69O23 | Fi56P20 |  | EST | Queue, ctg1243 |
| H42E07 | Fi56P20 |  | EST | Queue, ctg463 |
| B40G16 | Fi57K19 |  | EST | ctg1294, F, CEW 5-2 |
| H23M10 | Fi58L6 |  | EST | ctg1981, BSR 3-1, BSR 4-1, J |
| B18K03 | Fi58L6 |  | EST | ctg90, Queue |
| H27P14 | Fi58L6 |  | EST | ctg90, Queue |
| B44K21 | Fi58L6 |  | EST | Queue, ctg1890 |
| H49L04 | Fi58L6 |  | EST | Queue, ctg281 |
| B10B20 | Fi58L6 |  | EST | Queue, ctg612 |
| B37I13 | Fi65E19 | BI347339 | *Glycine max* myo-inositol-1-phosphate synthase (MI 1-P SYNTHASE) mRNA, complete cds. | ctg115, Pod mat 5-4, K |
| H26I22 | Fi65E19 | BI347339 | *Glycine max* myo-inositol-1-phosphate synthase (MI 1-P SYNTHASE) mRNA, complete cds. | Queue, ctg1300 |
| B31H04 | Fi65E19 | BI347339 | *Glycine max* myo-inositol-1-phosphate synthase (MI 1-P SYNTHASE) mRNA, complete cds. | Queue, ctg203 |
| B31G01 | Fi65E19 | BI347339 | *Glycine max* myo-inositol-1-phosphate synthase (MI 1-P SYNTHASE) mRNA, complete cds. | Queue, ctg218 |
| H21M23 | Fi65E19 | BI347339 | *Glycine max* myo-inositol-1-phosphate synthase (MI 1-P SYNTHASE) mRNA, complete cds. |  |
| H24L08 | FiS1A12 | BI119551 | *Glycine max* chalcone synthase (chs7) gene, complete cds. | Queue, ctg304 |
| H05G07 | FiS1A14 | BI119552 | EST | Queue, ctg1060 |
| H63N20 | FiS1A14 | BI119552 | EST | Queue, ctg1621 |
| H73G05 | FiS1A14 | BI119552 | EST | Queue, ctg1794 |
| H05F07 | FiS1A14 | BI119552 | EST | Queue, ctg1841 |
| B38M08 | FiS1A14 | BI119552 | EST | Queue, ctg206 |
| H63N22 | FiS1A14 | BI119552 | EST | Queue, ctg559 |
| H07B04 | FiS1A16 | BI119554 | *Glycine max* ADR12 mRNA | Queue, ctg1120 |
| B14I07 | FiS1A16 | BI119554 | *Glycine max* ADR12 mRNA | Queue, ctg1883 |
| B35O18 | FiS1A16 | BI119554 | *Glycine max* ADR12 mRNA | Queue, ctg287 |
| H07B04 | FiS1A17 | BI119550 | *Glycine max* actin (Soy57) gene, partial cds | Queue, ctg1120 |
| B35O18 | FiS1A17 | BI119550 | *Glycine max* actin (Soy57) gene, partial cds | Queue, ctg287 |
| E57A22 | FiS1A17 | BI119550 | *Glycine max* actin (Soy57) gene, partial cds |  |
| B32P09 | FiS1A17 | BI119550 | *Glycine max* actin (Soy57) gene, partial cds |  |
| H12A19 | FiS1A3 | BI273654 | EST | Queue, ctg1932 |
| B14O12 | FiS1A3 | BI273654 | EST | Queue, ctg434 |
| B14O15 | FiS1A3 | BI273654 | EST | Satt436c, D1AQ, ctg2183 |
| B32P09 | FiS1A4 | BI119562 | *Pisum sativum* ssa-15 mRNA for putative senescence-associated protein, complete cds. |  |
| H27M18 | FiS1A5 | BI119557 | *Glycine max* mRNA for cinnamic acid 4-hydroxylase (CYP73). | Queue, ctg2046 |
| H39D15 | FiS1A5 | BI119557 | *Glycine max* mRNA for cinnamic acid 4-hydroxylase (CYP73). | SCN 18-4, SCN 21-3, ctg155, E |
| H07B04 | FiS1A6 | BI119561 | *Glycine max* gene for ubiquitin, complete cds. | Queue, ctg1120 |
| H16B17 | FiS1B10 | BI119564 | Deoxychalcone synthesis (NAD(P)H dependent 6'-deoxychalcone synthase). | ctg1929, F |
| H28O23 | FiS1B10 | BI119564 | Deoxychalcone synthesis (NAD(P)H dependent 6'-deoxychalcone synthase). | Queue, ctg1780 |
| H64H17 | FiS1B10 | BI119564 | Deoxychalcone synthesis (NAD(P)H dependent 6'-deoxychalcone synthase). | Queue, ctg2144 |
| H45A23 | FiS1B10 | BI119564 | Deoxychalcone synthesis (NAD(P)H dependent 6'-deoxychalcone synthase). | Queue, ctg879 |
| H80F06 | FiS1B10 | BI119564 | Deoxychalcone synthesis (NAD(P)H dependent 6'-deoxychalcone synthase). |  |
| H27N04 | FiS1B10 | BI119564 | Deoxychalcone synthesis (NAD(P)H dependent 6'-deoxychalcone synthase). |  |
| H78G11 | FiS1B12 | BI119565 | *Solanum tuberosum* mRNA for putative membrane protein (poni2 gene). | ctg224, D1AQ |
| H67C16 | FiS1B12 | BI119565 | *Solanum tuberosum* mRNA for putative membrane protein (poni2 gene). | Queue, ctg1401 |
| H65B14 | FiS1B12 | BI119565 | *Solanum tuberosum* mRNA for putative membrane protein (poni2 gene). | Queue, ctg174 |
| B35O18 | FiS1B12 | BI119565 | *Solanum tuberosum* mRNA for putative membrane protein (poni2 gene). | Queue, ctg287 |
| B01H19 | FiS1B14 | BI119567 | *Glycyrrhiza echinata* mRNA for O-methyltransferase, complete cds. | ctg96, Queue |
| B35G12 | FiS1B14 | BI119567 | *Glycyrrhiza echinata* mRNA for O-methyltransferase, complete cds. | Satt466b, ctg504, O |
| H33N21 | FiS1B16 | BI119568 | seven in absentia-like protein | ctg176, G, SCN 15-1 |
| B13I24 | FiS1B16 | BI119568 | seven in absentia-like protein | Queue, ctg1242 |
| H13N16 | FiS1B16 | BI119568 | seven in absentia-like protein | Queue, ctg1533 |
| B38J19 | FiS1B16 | BI119568 | seven in absentia-like protein | Queue, ctg1683 |
| B38M08 | FiS1B16 | BI119568 | seven in absentia-like protein | Queue, ctg206 |
| B38J20 | FiS1B16 | BI119568 | seven in absentia-like protein | Queue, ctg356 |
| B48E15 | FiS1B16 | BI119568 | seven in absentia-like protein | Queue, ctg383 |
| H13N14 | FiS1B16 | BI119568 | seven in absentia-like protein | Queue, ctg829 |
| H24N16 | FiS1B16 | BI119568 | seven in absentia-like protein | Queue, ctg829 |
| H23L03 | FiS1B16 | BI119568 | seven in absentia-like protein | Queue, Satt621a, ctg667 |
| B03K04 | FiS1B2 | BI119569 | EST | ctg768, A1 |
| H11D11 | FiS1B2 | BI119569 | EST | pA878a, pA486d, ctg768, A1 |
| H43E20 | FiS1B2 | BI119569 | EST | Queue, pA023e, ctg1675 |
| H39O23 | FiS1B2 | BI119569 | EST | Satt131c, ctg91, G, SCN 17-4, SCN 18-5, SCN 19-4 |
| B38M08 | FiS1B4 | BI119570 | EST | Queue, ctg206 |
| B24B13 | FiS1C17 | BI119577 | EST | Queue, ctg567 |
| H17P03 | FiS1C22 | BI245396 | EST | ctg703, Sclero 3-19, Sclero 5-16, Sclero 6-13, O |
| B51H17 | FiS1C22 | BI245396 | EST | Queue, ctg210 |
| E48C13 | FiS1C22 | BI245396 | EST |  |
| E76J17 | FiS1C22 | BI245396 | EST |  |
| H21P11 | FiS1C3 | BI245397 | *Glycine max* sucrose synthase (SS) mRNA, complete cds. | ctg889, Queue |
| H14L10 | FiS1C3 | BI245397 | *Glycine max* sucrose synthase (SS) mRNA, complete cds. | Queue, ctg141 |
| H45O20 | FiS1C9 | BI245401 | *Medicago sativa* isoflavone-O-methytransferase mRNA, complete cds. | ctg61, D1BW |
| B03H05 | FiS1D1 | BI245402 | *Glycine max* peroxidase precursor (GMIPER1) mRNA, complete cds. | ctg2012, C2 |
| H65D13 | FiS1D1 | BI245402 | *Glycine max* peroxidase precursor (GMIPER1) mRNA, complete cds. | ctg2012, C2 |
| H08D19 | FiS1D1 | BI245402 | *Glycine max* peroxidase precursor (GMIPER1) mRNA, complete cds. | Queue, ctg1219 |
| H76H24 | FiS1D13 | BI245414 | EST | Queue, ctg759 |
| H26F23 | FiS1D24 | BI245409 | *Pisum sativum* brassinosteroid biosynthetic protein LKB (LKB) mRNA, complete cds. | ctg154, H |
| H26F07 | FiS1D24 | BI245409 | *Pisum sativum* brassinosteroid biosynthetic protein LKB (LKB) mRNA, complete cds. | Queue, ctg1020 |
| H34P05 | FiS1D24 | BI245409 | *Pisum sativum* brassinosteroid biosynthetic protein LKB (LKB) mRNA, complete cds. | Queue, ctg1043 |
| H27D03 | FiS1D24 | BI245409 | *Pisum sativum* brassinosteroid biosynthetic protein LKB (LKB) mRNA, complete cds. | Queue, ctg1627 |
| H26H01 | FiS1D24 | BI245409 | *Pisum sativum* brassinosteroid biosynthetic protein LKB (LKB) mRNA, complete cds. | Queue, ctg2045 |
| H26H03 | FiS1D24 | BI245409 | *Pisum sativum* brassinosteroid biosynthetic protein LKB (LKB) mRNA, complete cds. | Queue, ctg834 |
| H29L09 | FiS1D24 | BI245409 | *Pisum sativum* brassinosteroid biosynthetic protein LKB (LKB) mRNA, complete cds. |  |
| B35G05 | FiS1D3 | BI245410 | *Nicotiana. tabacum* mRNA for root-specific gene. | Queue, ctg1481 |
| H51K24 | FiS1D5 | BI245411 | EST | Queue, ctg1108 |
| H06N12 | FiS1D5 | BI245411 | EST | Queue, ctg1116 |
| B38M04 | FiS1D8 | BI118914 | *Solanum tuberosum* mRNA for plastidic ATP/ADP-transporter | ctg966, Queue |
| H42H06 | FiS1D8 | BI118914 | *Solanum tuberosum* mRNA for plastidic ATP/ADP-transporter | Queue, ctg486 |
| H16G17 | FiS1D8 | BI118914 | *Solanum tuberosum* mRNA for plastidic ATP/ADP-transporter | Satt130, Satt217d, CEW 8-1, ctg839, E |
| EMPTY | FiS1D8 | BI118914 | *Solanum tuberosum* mRNA for plastidic ATP/ADP-transporter |  |
| H43J22 | FiS1D8 | BI118914 | *Solanum tuberosum* mRNA for plastidic ATP/ADP-transporter |  |
| B48N15 | FiS1F1 | BI273644 | *Lupinus albus* mRNA for adenine nucleotide translocator. | Queue, ctg1217 |
| B38M08 | FiS1F8 | BI273649 | EST | Queue, ctg206 |
| B39I18 | FiS1F8 | BI273649 | EST | Queue, ctg828 |
| H44O11 | FiS1G15 | BI273650 | Alfalfa glucose-regulated endoplasmic reticular protein mRNA, complete cds. | ctg224, D1AQ |
| B14I07 | FiS1G16 | BI273651 | *Glycine max* PAL1 gene for phenylalanine ammonia lyase (EC 4.3.1.5). | Queue, ctg1883 |
| H15A05 | FiS1G17 | BI273652 | EST | Queue, ctg1961 |
| H36D08 | FiS1G17 | BI273652 | EST | Queue, ctg245 |
| H46N03 | FiS1G18 | BI273653 | *Glycine max* SbPRP1 gene encoding a proline-rich protein, complete cds. | ctg6, H |
| B35L22 | FiS1G18 | BI273653 | *Glycine max* SbPRP1 gene encoding a proline-rich protein, complete cds. | ctg933, Queue |
| H30C03 | FiS1G18 | BI273653 | *Glycine max* SbPRP1 gene encoding a proline-rich protein, complete cds. | K, Oil 1-2, ctg54 |
| B31H04 | FiS1G18 | BI273653 | *Glycine max* SbPRP1 gene encoding a proline-rich protein, complete cds. | Queue, ctg203 |
| H55O24 | FiS1G18 | BI273653 | *Glycine max* SbPRP1 gene encoding a proline-rich protein, complete cds. | Queue, ctg472 |
| H18C02 | FiS1H11 | BI273656 | *Pisum sativum* ubiquitin conjugating enzyme (UBC4), complete cds. | Queue, ctg1037 |
| H72A05 | FiS1H11 | BI273656 | *Pisum sativum* ubiquitin conjugating enzyme (UBC4), complete cds. | Queue, ctg1065 |
| B38M08 | FiS1H11 | BI273656 | *Pisum sativum* ubiquitin conjugating enzyme (UBC4), complete cds. | Queue, ctg206 |
| H33N21 | FiS1H23 | No Acc. # | *Glycine max* ascorbate peroxidase mRNA, complete cds | ctg176, G, SCN 15-1 |
| B35C12 | FiS1H23 | No Acc. # | *Glycine max* ascorbate peroxidase mRNA, complete cds | ctg688, K |
| H07B04 | FiS1H23 | No Acc. # | *Glycine max* ascorbate peroxidase mRNA, complete cds | Queue, ctg1120 |
| B13I24 | FiS1H23 | No Acc. # | *Glycine max* ascorbate peroxidase mRNA, complete cds | Queue, ctg1242 |
| B38J19 | FiS1H23 | No Acc. # | *Glycine max* ascorbate peroxidase mRNA, complete cds | Queue, ctg1683 |
| H52B23 | FiS1H23 | No Acc. # | *Glycine max* ascorbate peroxidase mRNA, complete cds | Queue, ctg1995 |
| B35O18 | FiS1H23 | No Acc. # | *Glycine max* ascorbate peroxidase mRNA, complete cds | Queue, ctg287 |
| B48E15 | FiS1H23 | No Acc. # | *Glycine max* ascorbate peroxidase mRNA, complete cds | Queue, ctg383 |
| H65E02 | FiS1H23 | No Acc. # | *Glycine max* ascorbate peroxidase mRNA, complete cds | Queue, ctg514 |
| H65E02 | FiS1H23 | No Acc. # | *Glycine max* ascorbate peroxidase mRNA, complete cds | Queue, ctg514 |
| B51J14 | FiS1H23 | No Acc. # | *Glycine max* ascorbate peroxidase mRNA, complete cds | Queue, ctg726 |
| H13N14 | FiS1H23 | No Acc. # | *Glycine max* ascorbate peroxidase mRNA, complete cds | Queue, ctg829 |
| H24N16 | FiS1H23 | No Acc. # | *Glycine max* ascorbate peroxidase mRNA, complete cds | Queue, ctg829 |
| H23L03 | FiS1H23 | No Acc. # | *Glycine max* ascorbate peroxidase mRNA, complete cds | Queue, Satt621a, ctg667 |
| H06I10 | FiS1H8 | BI245400 | *Zea mays* plasma membrane integral protein ZmPIP2-7 mRNA, complete cds. | ctg91, G, SCN 17-4, SCN 18-5, SCN 19-4 |
| B53F09 | FiS1H8 | BI245400 | *Zea mays* plasma membrane integral protein ZmPIP2-7 mRNA, complete cds. | Queue, ctg1347 |
| H24P07 | FiS1H8 | BI245400 | *Zea mays* plasma membrane integral protein ZmPIP2-7 mRNA, complete cds. | Queue, ctg1391 |
| B48B23 | FiS1H9 | BI273631 | Pea histone H2A mRNA | ctg9, Queue |
| H59N09 | FiS1H9 | BI273631 | Pea histone H2A mRNA | Queue, ctg1615 |
| H76L07 | FiS1H9 | BI273631 | Pea histone H2A mRNA |  |
| H15A05 | FiS1i10 | No Acc. # | *Cicer arietinum* mRNA for 20S proteasome beta subunit. | Queue, ctg1961 |
| H73G05 | FiS1i11 | BI273656 | *Stylosanthes humilis* cinnamyl alcohol dehydrogenase (CAD1) mRNA, complete cds | Queue, ctg1794 |
| H07B04 | FiS1i12 | BI273633 | *Oryza sativa* genomic DNA, chromosome 5, clone | Queue, ctg1120 |
| H25K14 | FiS1i13 | BI273634 | EST | K, ctg200 |
| H07B04 | FiS1i13 | BI273634 | EST | Queue, ctg1120 |
| H70L21 | FiS1i13 | BI273634 | EST | Queue, ctg1138 |
| H45I14 | FiS1i13 | BI273634 | EST | Queue, ctg223 |
| H61L12 | FiS1i13 | BI273634 | EST | Queue, ctg790 |
| H30H12 | FiS1i17 | BI273635 | EST | ctg801, G, Sclero 5-9 |
| H12A07 | FiS1i18 | No Acc. # | *Glycine max* mRNA for profilin, PRO1. | ctg991, Queue |
| H22G20 | FiS1i18 | No Acc. # | *Glycine max* mRNA for profilin, PRO1. | Oil 8-1, Sd wt 7-3, A1, ctg586 |
| H37G08 | FiS1i18 | No Acc. # | *Glycine max* mRNA for profilin, PRO1. | Queue, ctg1161 |
| H100o03 | FiS1i18 | No Acc. # | *Glycine max* mRNA for profilin, PRO1. |  |
| H33N21 | FiS1i19 | BI273655 | *Glycine max* ascorbate peroxidase mRNA, complete cds. | ctg176, G, SCN 15-1 |
| B13I24 | FiS1i19 | BI273655 | *Glycine max* ascorbate peroxidase mRNA, complete cds. | Queue, ctg1242 |
| H13N16 | FiS1i19 | BI273655 | *Glycine max* ascorbate peroxidase mRNA, complete cds. | Queue, ctg1533 |
| B38J19 | FiS1i19 | BI273655 | *Glycine max* ascorbate peroxidase mRNA, complete cds. | Queue, ctg1683 |
| B01B05 | FiS1i19 | BI273655 | *Glycine max* ascorbate peroxidase mRNA, complete cds. | Queue, ctg458 |
| H13N14 | FiS1i19 | BI273655 | *Glycine max* ascorbate peroxidase mRNA, complete cds. | Queue, ctg829 |
| H24N16 | FiS1i19 | BI273655 | *Glycine max* ascorbate peroxidase mRNA, complete cds. | Queue, ctg829 |
| H23L03 | FiS1i19 | BI273655 | *Glycine max* ascorbate peroxidase mRNA, complete cds. | Queue, Satt621a, ctg667 |
| H27O14 | FiS1i2 | BI273663 | EST |  |
| H70I24 | FiS1i22 | BI273638 | *Daucus carota* mRNA for AX110P. | Queue, ctg621 |
| B14I07 | FiS1i3 | BI273639 | EST | Queue, ctg1883 |
| H07D18 | FiS1i3 | BI273639 | EST | Queue, ctg781 |
| H60O14 | FiS1i6 | No Acc. # | *Glycine max* Williams 82 lipoxygenase mRNA, complete cds. | C2, CEW 3-1, ctg771 |
| H30G20 | FiS1i6 | No Acc. # | *Glycine max* Williams 82 lipoxygenase mRNA, complete cds. | ctg144, D1BW |
| B23D17 | FiS1i6 | No Acc. # | *Glycine max* Williams 82 lipoxygenase mRNA, complete cds. | ctg289, Sd wt 12-2, O |
| H23D11 | FiS1i6 | No Acc. # | *Glycine max* Williams 82 lipoxygenase mRNA, complete cds. | ctg386, SCN 22-3, Linolen 1-3, Oil 2-9, E |
| H41N24 | FiS1i6 | No Acc. # | *Glycine max* Williams 82 lipoxygenase mRNA, complete cds. | G, Sclero 5-9, ctg282 |
| H41P06 | FiS1i6 | No Acc. # | *Glycine max* Williams 82 lipoxygenase mRNA, complete cds. | Queue, ctg133 |
| B14F03 | FiS1i6 | No Acc. # | *Glycine max* Williams 82 lipoxygenase mRNA, complete cds. | Queue, ctg1447 |
| H46O24 | FiS1i6 | No Acc. # | *Glycine max* Williams 82 lipoxygenase mRNA, complete cds. | Queue, ctg1447 |
| H61A13 | FiS1i6 | No Acc. # | *Glycine max* Williams 82 lipoxygenase mRNA, complete cds. | Queue, ctg1645 |
| B04A07 | FiS1i6 | No Acc. # | *Glycine max* Williams 82 lipoxygenase mRNA, complete cds. | Queue, ctg1668 |
| B35O18 | FiS1i6 | No Acc. # | *Glycine max* Williams 82 lipoxygenase mRNA, complete cds. | Queue, ctg287 |
| B45H11 | FiS1i6 | No Acc. # | *Glycine max* Williams 82 lipoxygenase mRNA, complete cds. | Queue, ctg291 |
| H29A08 | FiS1i6 | No Acc. # | *Glycine max* Williams 82 lipoxygenase mRNA, complete cds. | Queue, ctg63 |
| H31N21 | FiS1i6 | No Acc. # | *Glycine max* Williams 82 lipoxygenase mRNA, complete cds. | Queue, ctg697 |
| H12E08 | FiS1i6 | No Acc. # | *Glycine max* Williams 82 lipoxygenase mRNA, complete cds. | Satt503, G, Sclero 5-9, ctg282 |
| B15A19 | FiS1i7 | BI273640 | EST | Queue, ctg1417 |
| B16B01 | FiS1i7 | BI273640 | EST | Queue, ctg1483 |
| H03L02 | FiS1i7 | BI273640 | EST | Queue, ctg1483 |
| H30M22 | FiS1i8 | BI273641 | EST | Queue, ctg1360 |
| H27O14 | FiS1i8 | BI273641 | EST |  |
| H74E06 | FiS1i9 | BI245403 | *Phaseolus* *acutifolius* alcohol dehydrogenase-1F mRNA, complete CDS. | ctg924, Queue |
| H77F19 | FiS1i9 | BI245403 | *Phaseolus* *acutifolius* alcohol dehydrogenase-1F mRNA, complete CDS. | Queue, ctg198 |
| H63N22 | FiS1i9 | BI245403 | *Phaseolus* *acutifolius* alcohol dehydrogenase-1F mRNA, complete CDS. | Queue, ctg559 |
| H45E21 | FiS1i9 | BI245403 | *Phaseolus* *acutifolius* alcohol dehydrogenase-1F mRNA, complete CDS. | Queue, pA257d, ctg338 |
| H21F06 | FiS1J1 | No Acc. # | EST | pA711c, pB208c, ctg1203, A2 |
| H47J16 | FiS1J15 | No Acc. # | EST | ctg913, Queue |
| H15I03 | FiS1J15 | No Acc. # | EST | Satt300b, ctg625, A1, SCN 18-1, Fe effic 5-1 |
| H45I14 | FiS1J16 | BI119556 | *Glycine max* nodulin 22 gene. | Queue, ctg223 |
| B35O18 | FiS1J16 | BI119556 | *Glycine max* nodulin 22 gene. | Queue, ctg287 |
| B09E12 | FiS1J16 | BI119556 | *Glycine max* nodulin 22 gene. | Queue, ctg39 |
| H61L12 | FiS1J16 | BI119556 | *Glycine max* nodulin 22 gene. | Queue, ctg790 |
| H26H03 | FiS1J16 | BI119556 | *Glycine max* nodulin 22 gene. | Queue, ctg834 |
| H68C24 | FiS1J17 | BI273660 | EST | Queue, ctg1020 |
| H26N07 | FiS1J17 | BI273660 | EST | Queue, ctg1389 |
| H63N20 | FiS1J17 | BI273660 | EST | Queue, ctg1621 |
| B48M16 | FiS1J17 | BI273660 | EST | Queue, ctg1696 |
| H26N06 | FiS1J17 | BI273660 | EST | Queue, ctg444 |
| H45C14 | FiS1J17 | BI273660 | EST | Queue, ctg52 |
| H63N22 | FiS1J17 | BI273660 | EST | Queue, ctg559 |
| H15I03 | FiS1J17 | BI273660 | EST | Satt300b, ctg625, A1, SCN 18-1, Fe effic 5-1 |
| H80P20 | FiS1J17 | BI273660 | EST |  |
| H39O23 | FiS1J18 | BI273661 | *Glycine max* mRNA for nodulin-26. | Satt131c, ctg91, G, SCN 17-4, SCN 18-5, SCN 19-4 |
| H17P03 | FiS1J2 | BI273662 | *Medicago sativa* mRNA for protein phosphatase 1, beta subunit. | ctg703, Sclero 3-19, Sclero 5-16, Sclero 6-13, O |
| H13K14 | FiS1J20 | No Acc. # | *Cicer arietinum* partial mRNA for putative water channel protein | Queue, ctg124 |
| B53F09 | FiS1J20 | No Acc. # | *Cicer arietinum* partial mRNA for putative water channel protein | Queue, ctg1347 |
| H24P07 | FiS1J20 | No Acc. # | *Cicer arietinum* partial mRNA for putative water channel protein | Queue, ctg1391 |
| H12B22 | FiS1J20 | No Acc. # | *Cicer arietinum* partial mRNA for putative water channel protein | Queue, ctg1765 |
| B38M08 | FiS1J20 | No Acc. # | *Cicer arietinum* partial mRNA for putative water channel protein | Queue, ctg206 |
| B53F20 | FiS1J20 | No Acc. # | *Cicer arietinum* partial mRNA for putative water channel protein | Queue, ctg316 |
| B09E12 | FiS1J20 | No Acc. # | *Cicer arietinum* partial mRNA for putative water channel protein | Queue, ctg39 |
| B08N01 | FiS1J20 | No Acc. # | *Cicer arietinum* partial mRNA for putative water channel protein | Queue, ctg760 |
| H76O12 | FiS1J20 | No Acc. # | *Cicer arietinum* partial mRNA for putative water channel protein | Queue, sct_065a, ctg470 |
| H26D04 | FiS1J20 | No Acc. # | *Cicer arietinum* partial mRNA for putative water channel protein | SCN 19-1, ctg292, Fe effic 1-1, Hrd Sd 1-1, SCN 3-1, SCN 9-2, Hrd Sd 1-2, Sd wt 4-5, Sucrose 1-3, A2, Oil 1-1 |
| B52F16 | FiS1J21 | No Acc. # | *Medicago sativa* eukaryotic translation initiation factor 5A-2 mRNA, complete cds. | Queue, ctg1314 |
| H26N07 | FiS1J21 | No Acc. # | *Medicago sativa* eukaryotic translation initiation factor 5A-2 mRNA, complete cds. | Queue, ctg1389 |
| H08I21 | FiS1J21 | No Acc. # | *Medicago sativa* eukaryotic translation initiation factor 5A-2 mRNA, complete cds. | Queue, ctg1550 |
| H26N06 | FiS1J21 | No Acc. # | *Medicago sativa* eukaryotic translation initiation factor 5A-2 mRNA, complete cds. | Queue, ctg444 |
| H45C14 | FiS1J21 | No Acc. # | *Medicago sativa* eukaryotic translation initiation factor 5A-2 mRNA, complete cds. | Queue, ctg52 |
| H15I03 | FiS1J21 | No Acc. # | *Medicago sativa* eukaryotic translation initiation factor 5A-2 mRNA, complete cds. | Satt300b, ctg625, A1, SCN 18-1, Fe effic 5-1 |
| B37I13 | FiS1J22 | BI273637 | EST | ctg115, Pod mat 5-4, K |
| H24L08 | FiS1J22 | BI273637 | EST | Queue, ctg304 |
| H25L21 | FiS1J22 | BI273637 | EST | Queue, ctg351 |
| H25K14 | FiS1J23 | No Acc. # | *Glycine max* farnesylated protein GMFP5 mRNA, partial cds. | K, ctg200 |
| H25B11 | FiS1J23 | No Acc. # | *Glycine max* farnesylated protein GMFP5 mRNA, partial cds. | Queue, ctg1664 |
| H25J14 | FiS1J23 | No Acc. # | *Glycine max* farnesylated protein GMFP5 mRNA, partial cds. | Queue, ctg1664 |
| H25D05 | FiS1J23 | No Acc. # | *Glycine max* farnesylated protein GMFP5 mRNA, partial cds. | Queue, ctg1840 |
| H45I14 | FiS1J23 | No Acc. # | *Glycine max* farnesylated protein GMFP5 mRNA, partial cds. | Queue, ctg223 |
| H70M18 | FiS1J23 | No Acc. # | *Glycine max* farnesylated protein GMFP5 mRNA, partial cds. | Queue, ctg364 |
| H11M05 | FiS1J23 | No Acc. # | *Glycine max* farnesylated protein GMFP5 mRNA, partial cds. | Queue, ctg549 |
| B52E15 | FiS1J24 | BI273675 | *Vigna radiata* beta galactosidase mRNA, complete cds. | Queue, ctg1921 |
| H39O23 | FiS1J3 | BI273665 | *Gallus gallus* leukemia/lymohoma related factor cLRF (LRF) mRNA, complete cds. | Satt131c, ctg91, G, SCN 17-4, SCN 18-5, SCN 19-4 |
| H08D05 | FiS1J7 | BI119563 | EST | ctg2029, Oil/Prot 1-1, Pod mat 7-2, A1, SCN 18-1 |
| H17P03 | FiS1J7 | BI119563 | EST | ctg703, Sclero 3-19, Sclero 5-16, Sclero 6-13, O |
| B54E07 | FiS1K10 | No Acc. # | *Mediacgo.sativa* mRNA for peroxidase 1A. | ctg893, Queue |
| H07B04 | FiS1K10 | No Acc. # | *Mediacgo.sativa* mRNA for peroxidase 1A. | Queue, ctg1120 |
| H07B04 | FiS1K17 | BI273671 | EST | Queue, ctg1120 |
| H57J19 | FiS1K17 | BI273671 | EST | Queue, ctg1820 |
| H07B04 | FiS1K18 | BI273672 | EST | Queue, ctg1120 |
| H07K13 | FiS1K18 | BI273672 | EST | Queue, ctg419 |
| H73K21 | FiS1K4 | BI273664 | Leghemoglobin [Psophocarpus tetragonolobus=winged-beans, nodule, mRNA, | ctg801, G, Sclero 5-9 |
| H50N07 | FiS1K4 | BI273664 | Leghemoglobin [Psophocarpus tetragonolobus=winged-beans, nodule, mRNA, | Queue, ctg2263 |
| H24L07 | FiS1K4 | BI273664 | Leghemoglobin [Psophocarpus tetragonolobus=winged-beans, nodule, mRNA, | Satt305a, ctg483, Sd abrt 1-8, C2 |
| H42K05 | FiS1K5 | BI273687 | *Glycine max* actin (Soy58) gene, partial cds. | Queue, ctg208 |
| H54N14 | FiS1K7 | BI273678 | *Arabidopsis thaliana* peroxisomal 3-keto-acyl-CoA thiolase 2 precursor (PKT2) mRNA, complete cds. | Queue, ctg1339 |
| B53F09 | FiS1K7 | BI273678 | *Arabidopsis thaliana* peroxisomal 3-keto-acyl-CoA thiolase 2 precursor (PKT2) mRNA, complete cds. | Queue, ctg1347 |
| B17O12 | FiS1M22 | BI273681 | *Oryza sativa* genomic DNA, chromosome 1, PAC clone:P0408C03, complete sequence. | ctg689, H |
| H33N21 | FiS1M24 | No Acc. # | *Solanum melongena* mRNA for QM family protein, complete cds. | ctg176, G, SCN 15-1 |
| H07B04 | FiS1M24 | No Acc. # | *Solanum melongena* mRNA for QM family protein, complete cds. | Queue, ctg1120 |
| B13I24 | FiS1M24 | No Acc. # | *Solanum melongena* mRNA for QM family protein, complete cds. | Queue, ctg1242 |
| B38J19 | FiS1M24 | No Acc. # | *Solanum melongena* mRNA for QM family protein, complete cds. | Queue, ctg1683 |
| H13N14 | FiS1M24 | No Acc. # | *Solanum melongena* mRNA for QM family protein, complete cds. | Queue, ctg829 |
| H24N16 | FiS1M24 | No Acc. # | *Solanum melongena* mRNA for QM family protein, complete cds. | Queue, ctg829 |
| H23L03 | FiS1M24 | No Acc. # | *Solanum melongena* mRNA for QM family protein, complete cds. | Queue, Satt621a, ctg667 |
| B53F09 | FiS1M4 | BI273682 | *Glycine max* putative water channel protein (Pip1) mRNA, complete cds. | Queue, ctg1347 |
| B17D02 | FiS1M6 | BI245393 | EST | Queue, ctg1056 |
| B50G06 | FiS1M6 | BI245393 | EST | Queue, pA338d, ctg1056 |
| B54E07 | FiS1N17 | BI273683 | EST | ctg893, Queue |
| H07B04 | FiS1N17 | BI273683 | EST | Queue, ctg1120 |
| B46H23 | FiS1N17 | BI273683 | EST | Queue, ctg876 |
| H58J12 | FiS1N17 | BI273683 | EST |  |
| H15I03 | FiS1N18 | BI119551 | chalcone synthase [soybeans, mRNA, 1119 nt]. | Satt300b, ctg625, A1, SCN 18-1, Fe effic 5-1 |
| H08D05 | FiS1N21 | BI273684 | EST | ctg2029, Oil/Prot 1-1, Pod mat 7-2, A1, SCN 18-1 |
| H17P03 | FiS1N21 | BI273684 | EST | ctg703, Sclero 3-19, Sclero 5-16, Sclero 6-13, O |
| H07B04 | FiS1N21 | BI273684 | EST | Queue, ctg1120 |
| B46H23 | FiS1N21 | BI273684 | EST | Queue, ctg876 |
| H58J12 | FiS1N21 | BI273684 | EST |  |
| B40C23 | FiS1N22 | BI273685 | EST | Queue, ctg1306 |
| B44I13 | FiS1N22 | BI273685 | EST | Queue, Satt410b, ctg767 |
| H13E15 | FiS1N3 | BI273686 | *Daucus carota* poly(A)-binding protein mRNA, complete cds. | Queue, ctg1238 |
| B40C23 | FiS1O1 | BI273676 | Phenylalanine ammonia-lyase [soybeans, mRNA, 1427 nt]. | Queue, ctg1306 |
| H23D11 | FiS1O7 | BI273688 | *Nicotiana tabacum* DNA-directed RNA polymerase IIa mRNA, complete cds. | ctg386, SCN 22-3, Linolen 1-3, Oil 2-9, E |
| H42N09 | FiS1O7 | BI273688 | *Nicotiana tabacum* DNA-directed RNA polymerase IIa mRNA, complete cds. | O, ctg1271 |
| H41P06 | FiS1O7 | BI273688 | *Nicotiana tabacum* DNA-directed RNA polymerase IIa mRNA, complete cds. | Queue, ctg133 |
| H53H09 | FiS1O7 | BI273688 | *Nicotiana tabacum* DNA-directed RNA polymerase IIa mRNA, complete cds. | Queue, ctg19 |
| B38M08 | FiS1O7 | BI273688 | *Nicotiana tabacum* DNA-directed RNA polymerase IIa mRNA, complete cds. | Queue, ctg206 |
| B53F20 | FiS1O7 | BI273688 | *Nicotiana tabacum* DNA-directed RNA polymerase IIa mRNA, complete cds. | Queue, ctg316 |
| H37L06 | FiS1O7 | BI273688 | *Nicotiana tabacum* DNA-directed RNA polymerase IIa mRNA, complete cds. | Queue, ctg387 |
| H45E21 | FiS1O7 | BI273688 | *Nicotiana tabacum* DNA-directed RNA polymerase IIa mRNA, complete cds. | Queue, pA257d, ctg338 |
| B53F09 | FiS2D22 | BM499231 | *Medicago truncatula* zinc transporter (ZIP) mRNA, complete cds. | Queue, ctg1347 |
| H24P07 | FiS2D22 | BM499231 | *Medicago truncatula* zinc transporter (ZIP) mRNA, complete cds. | Queue, ctg1391 |
| H08I21 | FiS2F14 | No Acc. # | *Glycine max* isoflavone synthase 1 (ifs1) mRNA, complete cds. | Queue, ctg1550 |
| B53F09 | FiS2F18 | BM499236 | *Glycine max* gene for ubiquitin, complete cds. | Queue, ctg1347 |
| H24P07 | FiS2F18 | BM499236 | *Glycine max* gene for ubiquitin, complete cds. | Queue, ctg1391 |
| H70A24 | FiS2H1 | No Acc. # | *Cypripedium arietinum* mRNA for class I type 2 metallothionein (clone: CanMT-2). | Queue, ctg778 |
| H45E21 | FiS2H1 | No Acc. # | *Cypripedium arietinum* mRNA for class I type 2 metallothionein (clone: CanMT-2). | Queue, pA257d, ctg338 |
| B19N19 | FiS2H22 | BM499239 | *Nicotiana attenuata* pathogen-inducible alpha-dioxygenase (PIOX_NICAT) mRNA, complete cds. | ctg108, C2 |
| B03K04 | FiS2H22 | BM499239 | *Nicotiana attenuata* pathogen-inducible alpha-dioxygenase (PIOX_NICAT) mRNA, complete cds. | ctg768, A1 |
| H42N09 | FiS2H22 | BM499239 | *Nicotiana attenuata* pathogen-inducible alpha-dioxygenase (PIOX_NICAT) mRNA, complete cds. | O, ctg1271 |
| B35O03 | FiS2H22 | BM499239 | *Nicotiana attenuata* pathogen-inducible alpha-dioxygenase (PIOX_NICAT) mRNA, complete cds. | Queue, ctg20 |
| B38M08 | FiS2H22 | BM499239 | *Nicotiana attenuata* pathogen-inducible alpha-dioxygenase (PIOX_NICAT) mRNA, complete cds. | Queue, ctg206 |
| H43E20 | FiS2H22 | BM499239 | *Nicotiana attenuata* pathogen-inducible alpha-dioxygenase (PIOX_NICAT) mRNA, complete cds. | Queue, pA023e, ctg1675 |
| H39O23 | FiS2H22 | BM499239 | *Nicotiana attenuata* pathogen-inducible alpha-dioxygenase (PIOX_NICAT) mRNA, complete cds. | Satt131c, ctg91, G, SCN 17-4, SCN 18-5, SCN 19-4 |
| H26F07 | FiS2H8 | BM499232 | *Glycine max* nodulin 22 gene. | Queue, ctg1020 |
| H68C24 | FiS2J8 | BM499234 | *Glycine max* nodulin (E27) | Queue, ctg1020 |
| B50N05 | FiS2J8 | BM499234 | *Glycine max* nodulin (E27) | Queue, ctg1752 |
| H80P20 | FiS2J8 | BM499234 | *Glycine max* nodulin (E27) |  |
| H59B21 | FiS2L2 | No Acc. # | *Vigna unguiculata* CPRD86 mRNA, partial cds. | Queue, ctg1125 |
| H45C14 | FiS2L2 | No Acc. # | *Vigna unguiculata* CPRD86 mRNA, partial cds. | Queue, ctg52 |
| H33G19 | FiS2L20 | No Acc. # | *Sesbania rostrata* mRNA for phosphate transporter (pt1 gene). | Queue, ctg1075 |
| H25L07 | FiS2L20 | No Acc. # | *Sesbania rostrata* mRNA for phosphate transporter (pt1 gene). | Queue, ctg1555 |
| H47G08 | FiS2L20 | No Acc. # | *Sesbania rostrata* mRNA for phosphate transporter (pt1 gene). | Queue, ctg1555 |
| H12A07 | FiS2L8 | BM499240 | EST | ctg991, Queue |
| H22G20 | FiS2L8 | BM499240 | EST | Oil 8-1, Sd wt 7-3, A1, ctg586 |
| H37G08 | FiS2L8 | BM499240 | EST | Queue, ctg1161 |
| H100o03 | FiS2L8 | BM499240 | EST |  |
| B32P09 | FiS2N20 | BM499228 | *Vigna unguiculata* phospholipase D mRNA, complete cds. | |
| H48D17 | FiS2N4 | BM499242 | EST | Queue, ctg1095 |
